# β-Nicotinamide Mononucleotide Reduces Oxidative Stress and Improves Steroidogenesis in Granulosa Cells Associated with Sheep Prolificacy via Activating AMPK Pathway

**DOI:** 10.3390/antiox14010034

**Published:** 2024-12-30

**Authors:** Yu Cai, Hua Yang, Hui Xu, Shanglai Li, Bingru Zhao, Zhibo Wang, Xiaolei Yao, Feng Wang, Yanli Zhang

**Affiliations:** Jiangsu Livestock Embryo Engineering Laboratory, College of Animal Science and Technology, Nanjing Agricultural University, Nanjing 210095, China; 2021205019@stu.njau.edu.cn (Y.C.); yanghua004@njau.edu.cn (H.Y.); 2022105004@stu.njau.edu.cn (H.X.); 2021105035@stu.njau.edu.cn (S.L.); t2022070@njau.edu.cn (B.Z.); 2020205010@njau.edu.cn (Z.W.); yaoxiaolei@njau.edu.cn (X.Y.); caeet@njau.edu.cn (F.W.)

**Keywords:** β-nicotinamide mononucleotide, granulosa cells, oxidative stress, apoptosis, steroidogenesis, AMPK pathway

## Abstract

Oxidative stress is a significant factor in the death of granulosa cells (GCs), leading to follicular atresia and consequently limiting the number of dominant follicles that can mature and ovulate within each follicular wave. Follicular fluid contains a diverse array of metabolites that play crucial roles in regulating GCs’ proliferation and oocyte maturation, which are essential for follicle development and female fertility. However, the mechanisms behind metabolite heterogeneity and its effects on GCs’ function remain poorly understood. Here, we identified elevated nicotinamide levels in the follicular fluid of high-prolificacy sheep, correlated with oxidative stress in GCs, by an integrated analysis. In vitro experiments demonstrated that supplementation with β-nicotinamide mononucleotide (NMN) significantly increased the levels of nicotinamide adenine dinucleotide (NAD+) and adenosine triphosphate (ATP) in GCs. NMN treatment effectively reduced Lipopolysaccharide (LPS)-induced apoptosis and mitigated mitochondrial dysfunction, while also decreasing the production of reactive oxygen species (ROS), thereby enhancing the activity of the antioxidant defense system. Importantly, NMN treatment improved the impairments in steroid hormone levels induced by LPS. Mechanistically, the protective effects of NMN against GCs function were mediated via the AMPK/mTOR pathway. Collectively, our findings elucidate the metabolic characteristics associated with sheep prolificacy and demonstrate that NMN effectively protects GCs from LPS-induced dysfunction and enhances ovarian responsiveness via the AMPK/mTOR pathway. These findings also position NMN as a potential novel metabolic biomarker in enhancing ovarian function.

## 1. Introduction

Reproductive traits are critical economic indicators in livestock, providing valuable insights into animal reproductive physiology and forming the theoretical basis for enhancing reproductive efficiency in animal breeding. Metabolomics has emerged as a powerful tool for predicting fecundity traits in molecular breeding. For example, the metabolic profile of follicular fluid (FF) has been identified as a source of key biomarkers associated with *human* infertility [1]. However, its relevance extends beyond *human* fertility to livestock breeding. In particular, studying FF is crucial in livestock, as it provides insights into *cattle* fertility [2]. Metabolites in follicular fluid and serum from *Holstein cows* have been shown to predict genetic merit for fertility and identify potential biomarkers for improving dairy *cow* fertility [2,3,4]. Similarly, the metabolomic profiles of FF in *goats* have been analyzed to identify biomarkers related to oocyte competence [5]. These studies underscore the broader potential of metabolomics in enhancing reproductive efficiency and advancing our understanding of reproductive physiology in livestock. *Hu sheep*, known for their year-round estrus and high prolificacy, serve as an exemplary model for regenerative medicine and tissue engineering [6], but also for reproduction and pregnancy disorders [7,8]. FF plays a vital physiological role as a nutrient reservoir for both oocytes and granulosa cells (GCs), supporting the maturation of ovarian follicles [9,10]. Additionally, metabolites in FF play a crucial role in signaling oocyte maturation and regulating the surrounding GCs [11]. The selection of dominant follicles is a critical event in follicular development, marking the transition toward ovulation [12]. This process is characterized by rapid GCs proliferation, which is essential for both follicle maturation and overall female fertility [13]. As follicles grow, both oocyte competence and the composition of FF change, reflecting the dynamic biochemical environment within the follicle [14]. Metabolic changes in the ovine FF are closely associated with follicular growth and maturation [15], and they influence the quality of both oocytes and GCs [14]. The proliferation and differentiation of GCs are indispensable for successful follicle development; however, oxidative stress-induced GCs death can lead to follicular atresia, negatively impacting ovulation and litter size [16]. Steroidogenesis, the process by which cholesterol is converted into steroid hormones (e.g., estrogen and progesterone) within theca and granulosa cells [17], is regulated by key enzymes, such as P450 side-chain cleavage (P450 scc), 3β-hydroxysteroid dehydrogenase (3β-HSD), and Cytochrome P450, family 19, subfamily a, polypeptide 1 (CYP19A1) [18]. Furthermore, Phosphatidylinositol 3-kinase/ RAC-alpha serine/threonine-protein kinase (PI3K/AKT) and extracellular regulated protein kinases (ERK1/2) pathways have been implicated in steroidogenesis [19,20]. Activation of mechanistic target of rapamycin kinase (mTOR) signaling is required for the follicle stimulating hormone (FSH)-mediated induction of several follicular differentiations [21]. However, the potential mechanisms and specific pathways by which FF metabolites affect GCs apoptosis and steroidogenesis have not been elucidated.

Nicotinamide, an essential antioxidant in *human* follicular fluid [22], serves as the precursor to β-nicotinamide mononucleotide (NMN) [23]. NMN is subsequently converted to nicotinamide adénine dinucléotide (NAD) in vivo, emphasizing the importance of nicotinamide in cellular health by various physiological processes, including energy metabolism, DNA repair, cell signaling, and regulation of oxidative stress [24]. By facilitating the availability of NAD, nicotinamide plays a crucial role in mitochondrial rejuvenation, anti-inflammatory, and anti-apoptotic pathways [25]. When reoxidized by O2 in mitochondria by the electron transport chain in the inner mitochondrial membrane, the reduced NADs lead to the production of adenosine triphosphate (ATP). Oxidation is catalyzed in several steps to release the energy gradually [26]. NMN increases NAD+ and ATP levels and exerts anti-apoptosis and anti-inflammatory activities, accompanied with mitochondrial oxidative stress and DNA damage in the MH-S cell line and mice [27,28]. Notably, NMN, particularly its active β-isomer, has been shown to mitigate oxidative stress, inflammation, and apoptosis through mechanisms involving mitochondrial regeneration and modulation of key proteins, such as sirtuin-1 (SIRT1) and Nicotinamide mononucleotide adenylyltransferase (NMNAT) protein [25]. Reactive oxygen species (ROS) are naturally products of normal metabolic processes, including reproductive physiology such as follicular growth, ovulation, and luteal function. However, excessive ROS overwhelm the antioxidant defense, causing oxidative stress and impairing oocyte quality, follicular development, and overall female fertility [29]. Research indicates that NMN supplementation has significant health benefits in reproductive contexts. For instance, NMN treatment has been shown to rejuvenate oocyte quality in aged mice, thereby restoring fertility [30]. Furthermore, NMN supplementation enhances the ovulation of aged oocytes and improves their meiotic competency and fertilization ability in naturally aged mice [31]. Although several pathways influenced by NMN supplementation, such as SIRT1/PGC-1α pathway in an epileptic *mouse* model [32], Wnt/β-catenin pathway in *mice* intestinal stem cells [33] and mTOR pathway in hepatocellular carcinoma [34], have been identified, the underlying mechanisms remain incompletely understood. Furthermore, postpartum uterine infections in dairy *cattle* elevate lipopolysaccharide (LPS) levels, activating immune responses in ovarian GCs and triggering pro-inflammatory cytokine release, which disrupts follicular growth, oocyte maturation, and ovarian function [35]. Exposure of LPS to *bovine* granulosa cells reduced estradiol levels [36,37]. Additionally, NMN has been demonstrated to alleviate LPS-induced inflammatory and oxidative injuries in the neural cells of a septic *mice* model [38], as well as to mitigate acute lung injury through the suppression of inflammation, oxidative stress [39] and apoptosis [40]. Energy depletion in ovarian follicles triggers GCs apoptosis, a hallmark of follicular atresia. To simulate follicular stress responses, we established in vitro models of oxidative stress and inflammation using LPS to treat *sheep* GCs [41]. Given these findings, it is imperative to identify effective strategies to improve granulosa cell dysfunction induced by LPS, which is critical for follicular maturation and ovulation.

In this study, we performed dominant follicle fluid metabolic and RNA-seq analyses to determine the involvement of nicotinamide in *sheep* prolificacy. Our study hypothesizes that NMN may play a protective role in mitigating oxidative stress and GCs damage induced by LPS in *sheep*, thereby enhancing follicular development and reproductive health. The objectives of this research are to explore the role of NMN in mitigating oxidative stress and apoptosis in GCs, investigate its effects on steroidogenesis, and provide new insights into the regulatory mechanisms underlying prolificacy.

## 2. Materials and Methods

### 2.1. Animals and Ethics Statement

*Hu sheep* were raised at Jiangsu Qianbao Animal Husbandry Co., Ltd., (Yancheng, China) under similar conditions, with free access to food, water, and natural lighting. Eight non-pregnant multiparous ewes (3.5 ± 0.64 years old) with an identical litter size based on three lambing records were selected and divided into two groups. The highly prolific group (HP) consisted of *sheep* with the genotypes FecB^BB^/BMP15^AA^/GDF9^BB^ [42], each with identical litter size records of three (*n* = 4). The low prolific group (LP) included *sheep* with the genotypes FecB^B+^/BMP15^BB^/GDF9^AA^ [43], each with identical litter size records of one (*n* = 4). The genotyping of FecB, GDF9 and BMP15, and the ovarian morphological characteristics of experimental Hu *sheep* are shown in Figure S1. The experimental *sheep* weight and growth traits, including body length, chest circumference, pipe circumference, and shiri wide data, are measured statistically.

### 2.2. Estrus Cycle

Synchronous estrus was established as described previously in 2018 [44]. The entire estrus cycle, including proestrus, estrus, diestrus, and late estrus, was identified using the Giemsa method through vaginal smears (Figure S1G). The RAM test was used to determine the estrous status. The experimental *sheep* were slaughtered within 12 h of estrus. The ewes were slaughtered following standard and ethical procedures in an approved slaughterhouse, ensuring minimal stress and proper handling throughout the process. Venous blood samples were collected throughout the estrous cycle and before slaughter for testing hormonal levels. Briefly, blood sampling began on Day 1 of the first estrus and continued throughout the cycle (Days 1, 2, 3, 4, 7, 11, and 15). Blood was collected at specific time points to track hormonal variations during the estrous cycle. Intensive blood collection was performed during the second estrus for 150 min, with samples taken every 30 min (0, 30, 60, 90, 120, 150 min). Samples were collected in heparinized tubes, left at 37 °C for 30 min, and centrifuged at 3000 rpm for 10 min to separate plasma. Plasma was immediately frozen in liquid nitrogen and stored at −80 °C. Ovaries from high prolific (HP) and low prolific (LP) Hu *sheep* were collected and trimmed of fat. A total of twenty follicles measuring 3–5 mm were mechanically isolated using watchmaker forceps. For follicular granulosa cells, 10^3^–10^4^ cells were collected for smart-seq, and 200 uL follicular fluid was used for metabolic sequencing. The samples were not pooled. All experimental procedures, including animal care, were approved by the Institutional Animal Care and Use Committee of Nanjing Agricultural University (SYXK2022–0031). All methods and experimentations were performed in accordance with the relevant guidelines and regulations.

### 2.3. Hematoxylin and Eosin (H&E) Staining on Ovary

To clarify the ovarian morphology of Hu *sheep* with different prolificacies, ovaries were collected for staining. All collected ovaries were fixed in 4% paraformaldehyde (PFA) for at least 24 h for histological evaluation using established protocols [43]. Briefly, the ovaries were then transferred to 70% ethanol, embedded in paraffin wax, and serial-sectioned (5 μm) using a microtome. The serial sections were mounted on glass slides and stained with hematoxylin and eosin. All sections were examined with a light microscope (Nikon, Tokyo, Japan).

### 2.4. Follicular Fluid Metabolomic Analysis

Ovarian follicles with a 3–5 mm diameter were mechanically isolated using watchmaker forceps. After isolation, follicular fluid was drawn with a syringe into 1.5 mL Eppendorf tubes and resuspended with 500 μL of PBS. The samples were centrifuged at 1500 rpm, 4 °C for 5 min into pellet granulosa cells (GCs). The supernatant was then quickly frozen in liquid nitrogen for metabolomics analysis and labeled as HP_B_FF-1/2/3/4 and LP_B_FF-1/2/3/4. For cell precipitates, an appropriate amount of lysis buffer was added on ice, followed by freezing in liquid nitrogen for smart-seq analysis. Using fine forceps and a needle, the theca cells (TCs) were gently scraped or teased out from the surrounding theca layer and the basal membrane for RNA-seq.

#### 2.4.1. Follicular Fluid Preparation and Metabolomic Detection

A total of 100 μL of supernatant from each sample was transferred to 2 mL Eppendorf tubes and mixed with 1 mL of pre-cooled methanol by vigorous vortexing. The mixture underwent 35 Hz grinding for 4 min and ultrasonication in an ice water bath for 5 min (repeated 3 times). After standing at −40 °C for one hour, samples were centrifuged at 4 °C, 12,000 rpm for 15 min. From the supernatant, 100 μL was transferred to fresh 1.5 mL tubes. For quality control (QC) samples, 20 μL from each sample was combined, evaporated in a vacuum concentrator, and treated with 30 μL of Methoxyamination hydrochloride (20 mg/mL in pyridine) at 80 °C for 30 min. Then, 40 μL of Bis(trimethylsilyl)trifluoroacetamide (BSTFA) reagent [1% Trimethylchlorosilane (TMCS), *v*/*v*] was added and incubated at 70 °C for 1.5 h. After cooling to room temperature, 5 μL of fatty acid methyl ester (FAMEs) in chloroform was added to the QC sample. All samples were analyzed using gas chromatography coupled with time-of-flight mass spectrometry (GC-TOF-MS; Allwegene Technology Co., Ltd., Beijing, China).

#### 2.4.2. Differential Metabolites Identification

Raw data analysis, including peak extraction, baseline adjustment, deconvolution, alignment, and integration, was performed using Chroma TOF software (V 4.3x, LECO). The LECO-Fiehn Rtx5 database was used to identify metabolites by matching mass spectra and retention indices. Peaks detected in fewer than half of the quality control (QC) samples or with a relative standard deviation (RSD) greater than 30% were excluded. Features with an RSD exceeding 30% were also omitted from subsequent analysis. The resulting three-dimensional dataset, comprising peak numbers, sample names, and normalized peak areas, was analyzed using the R package ‘meta’ for principal component analysis (PCA) and orthogonal projections to latent structures-discriminant analysis (OPLS-DA). Based on the OPLS-DA projections, a loading plot was generated to illustrate variable contributions to group differences. To refine this analysis, variables with a first principal component VIP value exceeding 1.0 were initially identified as altered metabolites. Remaining variables were assessed using Student’s *t*-test (Q-value > 0.05), discarding those showing no significance between groups. Additionally, commercial databases, such as KEGG (http://www.kegg.jp, accessed on 10 March 2023) and MetaboAnalyst (http://www.metaboanalyst.ca/, accessed on 10 March 2023), were consulted to identify metabolite pathways.

### 2.5. RNA-Seq and Bioinformatics Analysis

GCs were collected from 3–5 mm follicles, lysed in buffer, and frozen in liquid nitrogen for smart-seq detection. TCs were collected from the follicular basement membrane and similarly frozen for RNA-seq analysis. Total RNA was extracted using the Trizol reagent kit (Invitrogen, Carlsbad, CA, USA, 15596026), treated with RNase-free DNase I (Takara, Kusatsu, Japan), and assessed for integrity on 1% agarose gels. RNA quantity and quality were further evaluated using the Agilent 2100 Bioanalyzer (Agilent Technologies, Santa Clara, CA, USA) and NanoDrop spectrophotometer (Thermo Scientific, San-Antonio, DE, USA), respectively. RNA samples meeting quality standards were selected for sequencing and qPCR validation. Initially, eight Hu *sheep* were selected for the experiment, and sequencing was performed with four samples from each group. However, due to considerable individual variability, particularly observed during the PCA clustering, three of the groups showed better clustering and consistency. Twelve cDNA libraries were constructed to identify ovarian mRNAs in Hu *sheep* with high and low prolificacy, designated as HP_B_GCs-1/2/3, LP_B_GCs-1/2/3, HP_B_TCs-1/2/3, and LP_B_TCs-1/2/3.

Libraries were prepared using the NEBNext^®^ Ultra™ RNA Library Prep Kit for Illumina^®^ (NEB, Rowley, MA, USA) and sequenced on an Illumina Novaseq 6000 platform by Beijing Allwegene Technology Company (Beijing, China), and they generated paired-end 150 bp reads. Clean reads were obtained by removing adapters, poly-N sequences, and low-quality reads from raw data. Clean reads were aligned to the *sheep* Oar_Rambouillet_v1.0 genome (https://www.ncbi.nlm.nih.gov/datasets/genome/GCF_002742125.1/-, accessed on 10 January 2023) with HTSeq version 0.5.4 p3, and gene expression levels were estimated as Fragments Per Kilobase of transcript per Million fragments mapped (FPKM). Differential expression analysis across groups was conducted using the DESeq R package (version 1.10.1). Genes with an adjusted *p*-value < 0.05 were considered differentially expressed. Gene Ontology (GO) enrichment analysis of differentially expressed genes (DEGs) utilized the GOseq R package (version 4.3.3), which employs the Wallenius non-central hypergeometric distribution [45]. Statistical enrichment of DEGs in KEGG pathways was assessed using KOBAS software (version 3.0) [46].

### 2.6. Ovarian Granulosa Cells Culture and Treatments

Hu *sheep* ovaries were collected from a local abattoir in Taicang, Jiangsu, China (121°10′ E, 31°45′ N) during the breeding season (October to January). GCs were isolated and cultured as previously described [47]. Briefly, ovaries were immediately transported to the laboratory within two hours and rinsed three times with sterile 1× DPBS containing 1% streptomycin. Granulosa cells were aspirated from healthy follicles of medium size (2–5 mm) using a 10 mL syringe with a needle. The cells and follicular fluid were centrifuged at 1500 rpm for 15 min, washed with 1× DPBS, and then seeded in a T25 culture flask in Dulbecco’s modified Eagle’s medium/nutrient mixture F-12 (DMEM/F12, Gibco, Carlsbad, CA, USA) supplemented with 10% fetal bovine serum (FBS, Gibco) and 1× antibiotic/antimycotic (Gibco). Cells were cultured in a humidified atmosphere of 5% CO^2^ at 37 °C. Once near confluence, cells were trypsinized with 0.25% trypsin (Gibco, 25200056) for 1 min, resuspended in serum-free cryopreservation medium (New Cell, Suzhou, China, C40100), and stored in liquid nitrogen. The purity of granulosa cells was verified by LHR and FSHR staining, as shown in the Supplemental Figure S1. For cell treatments, GCs were seeded into different plates: 6 wells (1 × 10^6^ cells/well) for steroidogenesis, 12 wells (5 × 10^5^ cells/well) for RNA and protein collection, 24 wells (1 × 10^5^ cells/well) for immunofluorescence and proliferation, and 96 wells (1 × 10^4^ cells/well) for the CCK-8 assay in culture medium [46]. After 60–70% confluence, NMN (Selleck, Shanghai, China, S5259) and LPS (BS904-10 mg, Biosharp, Nanjing, China) and AMPK inhibitor Compound C (HY-13418A, MCE, Shanghai, China) treatments or co-treatments were dissolved by ultrapure water after sterilization and were used to treat GCs. Water was also added to the control group in the culture medium, which was not exposed to the NMN and LPS vehicle. The concentrations of NMN (40 μM, 100 μM, 200 μM, 400 μM), LPS (0, 1, 10, 80 μg/mL) and Compound C (10 nM) were used in this study. The concentration was selected based on the protocols and previous studies, also supported by the CCK8 assay; 100 μM NMN and 80 μg/mL of LPS were the optimal conditions for subsequent experiments.

### 2.7. Hormone Determination

*Sheep* blood was collected and plasma was obtained to detect the concentrations of LH (DRE-S9371c), FSH (DRE-S9373c), E2 (DRE-S9362c), P4 (DRE-S0512c), FS (DRE-S9369c), and GnRH (DRE-S0528c) using ELISA Kits (Kmales Biological Technology Co., Ltd., Shanghai, China), following the manufacturer’s instructions. The supernatant of the cell culture medium collected after treatment with LPS, NMN, and NMN + LPS for 24 h was measured using an enzyme-linked immunosorbent assay following the manufacturer’s instructions (E2, Angle Gene, Nanjing, China, cat: ANG-E211213S; P4, Angle Gene, Nanjing, China, cat: ANG-E134215S). The mean optical density (O.D.) value for each standard and sample was determined using a standard microplate reader (SPARK, Tecan, Grödig, Austria).

### 2.8. Immunofluorescence

Immunofluorescence analysis was performed as described previously [48]. GCs were seeded into 24 wells (1 × 10^5^ cells/well) and cultured for 48 h. Rabbit anti-LHR (bs-6431R, 1:500 dilution, Bioss, Beijing, China) and anti-FSHR (bs-20658R, 1:500 dilution, Bioss, Beijing, China), and CoraLite 594-conjugated goat anti-rabbit IgG (1:500 dilution) were used as primary and secondary antibodies, respectively. Nuclei were stained with DAPI and examined using an LSM 710 laser scanning confocal microscope (Carl Zeiss, Oberkochen, Germany).

### 2.9. Mitochondrial Content and Membrane Potential (Δψm) Detection

MitoTracker Green probe staining was used to evaluate the mitochondrial content. The GCs were cultured in 12-well plates. After the treatment, the cells were incubated with 100 nm Mito-Tracker Green (Beyotime, Shanghai, China) at 37 °C for 40 min. Subsequently, PBS was used to wash the cells and remove the Mito-Tracker Green. Finally, the fresh cell culture solution preheated to 37 °C was added to observe the mitochondria using an LSM 710 laser scanning confocal microscope (Carl Zeiss, Oberkochen, Germany).

The Δψm was determined by the Mitochondrial Membrane Potential Assay Kit (JC-1, abs50016, absin, Shanghai, China). Specifically, the cells were incubated in the 12-well plates, the original cell culture medium was removed, and 1 mL new cell culture medium and 1 mL JC-1 working solution were added and incubated at 37 °C for 20 min. Then, the cells were rinsed twice with JC-1 buffer (1×), and after washing, 2 mL cell culture medium was added. Finally, the laser scanning confocal microscope was used to detect the fluorescence levels of the JC-1 polymers and monomers. ImageJ 1.42 was used to analyze the relative ratio of the red fluorescence to the green fluorescence.

### 2.10. ROS Measurement

The treated GCs were incubated with 10 μM dichlorofluorescein diacetate (DCFH-DA) (Solarbio, Beijing, China) at 37 °C for 20 min. Following three washes with DMEM/F12 to remove the extracellular DCFH-DA, ROS images were obtained using a laser scanning confocal microscope. ImageJ 1.42 (developed by Wayne Rasband, MD, USA) was utilized to measure the fluorescence levels of intracellular ROS.

### 2.11. Enzyme Activity Determination

The supernatant of the cell culture medium collected after being treated with LPS, NMN, and NMN + LPS for 24 h was collected. According to previous studies [49], the activities of superoxide dismutase (SOD), catalase (CAT), total antioxidant capacity (T-AOC), and malondialdehyde (MDA) in *sheep* GCs were detected using the corresponding detection kits from Angle Gene (SOD: YH1202; CAT: YH1208; T-AOC: YH1246; MDA: YH1217).

### 2.12. ATP and NAD+ Concentration Detection

According to the instructions, the ATP and NAD+ contents in *sheep* GCs stimulated with LPS, NMN, and LPS+NMN for 24 h were measured using ATP content detection kits (Angle Gene, Nanjing, China, cat: XH4001) and the NAD+/NADH assay kit (Angle Gene, cat: CF1010, Nanjing, China). The protein concentrations were quantified using a BCA quantification kit (Thermo, cat: 23228, 23224, Rockford, IL, USA).

### 2.13. Flow Cytometry and Cell Proliferation Analysis

GCs were cultured in 6-well culture plates; after the treatment, the cells were collected by 0.25% trypsin without ethylenediamine tetra acetic acid (EDTA) (Gibco, Grand Island, NY, USA). Cell apoptosis was analyzed by flow cytometry (BD Biosciences, Franklin Lakes, NJ, USA) using an annexin V-fluorescein isothiocyanate/propidium iodide apoptosis detection kit (Vazyme, Nanjing, China). All data were analyzed using FlowJo software 10.8.1. Cell proliferation was analyzed using CCK8 (KeyGen, Nanjing, China) and EdU incorporation assays (KeyGen, Nanjing, China), as described previously [48].

### 2.14. qRT-PCR Analysis

Total RNA extraction and cDNA synthesis were performed as described previously [48]. All qRT-PCR reactions were performed in the QuantStudioTM 5 system using SYBR Green master mix (cat. Q711, Vazyme, Nanjing, China), following the manufacturer’s instructions. Beta-actin (ACTB) was used as internal controls. Mutations in key genes, such as BMPR-IB, GDF9, and BMP15, were detected using PCR-SSCP and PCR-RFLP techniques. Primer sequences of PCR for genotype identification are listed in Table S1. Primer sequences of qRT-PCR are listed in Table S2. The 2^–ΔΔCT^ method was used to analyze relative expression levels.

### 2.15. Western Blot Analysis

Western blot was performed according to our previously described methods with a slight modification [44]. Briefly, the dilution ratio of primary antibodies was listed in Table S3, while goat anti-rabbit IgG and goat anti-mouse IgG (Beyotime Biotechnology, Shanghai, China) were used as the secondary antibodies. Immunoreactions were visualized using an enhanced chemiluminescence detection system (Fujifilm, Tokyo, Japan). The protein band intensity was quantified using ImageJ software 1.42.

### 2.16. Statistical Analysis

For Hu *sheep* experimentations, data are presented as means ± standard deviation based on four independent experiments (n = 4). Non-parametric statistic tests were used to compare the differences between two groups. For the cell culture experimentations, at least three cell batches were performed, with three technical and biological repetitions to ensure reproducibility. Data are presented as means ± standard error of the mean based on three independent experiments. All data were normally distributed, and variance was similar between the statistically compared groups. Statistical analyses were performed using GraphPad Prism 8.2 (GraphPad, San Diego, CA, USA) and SPSS software, version 24.0. (IBM, Armonk, New York, NY, USA). A Student’s *t*-test was used to compare the differences between two groups. Multiple comparisons involved three or more groups.

## 3. Results

### 3.1. Blood Steroid Hormones Including E2 and P4 Increased in High-Prolific Hu Sheep

**Figure 1:**
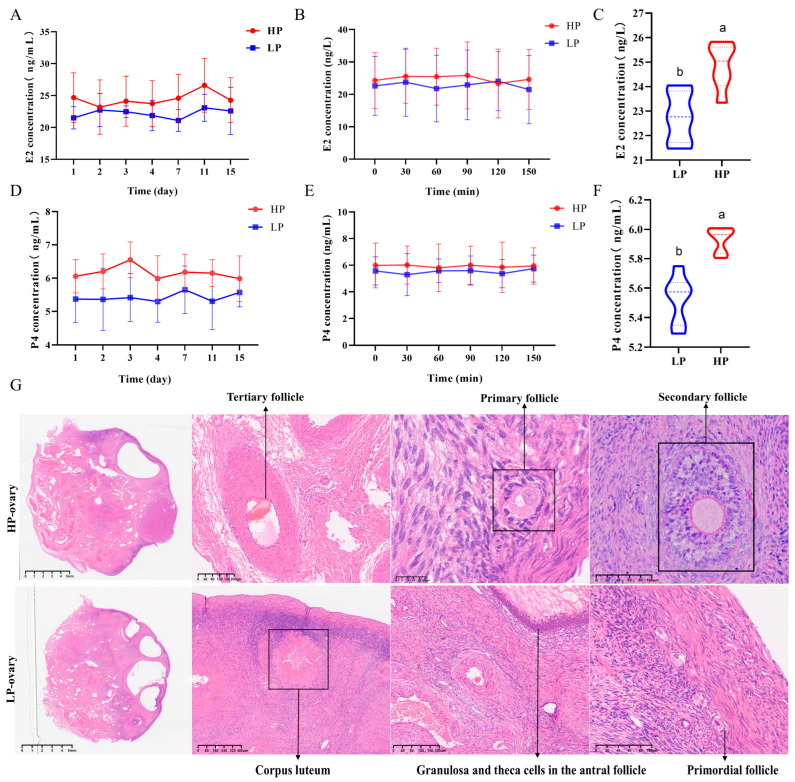
Blood biochemical and ovary morphology of *sheep* with different prolificacies. The estrogen (E2) concentration in the blood of Hu *sheep* with high and low prolificacy: (**A**) during the estrus cycle; (**B**) within 2.5 h before slaughter at the presumed estrus moment; (**C**) average in the estrus of Hu *sheep* with high and low prolificacy. The progestogen (P4) concentration in the blood of Hu *sheep* with high and low prolificacy: (**D**) during the estrus cycle; (**E**) within 2.5 h before slaughter at the presumed estrus moment; (**F**) average in the estrus of Hu *sheep* with high and low prolificacy. Time points in the graphs are relative to the first estrus cycle (Day 0 = estrus onset), and minutes refer to the intensive blood collection period during the second estrus (n = 4 for each LP or HP group). (**G**) HE staining of ovary tissue of Hu *sheep* with high (above, HP-ovary) and low (below, LP-ovary) prolificacy. Values (lowercase letter a or b) without a common superscript differ (*p* < 0.05).

High-prolific (FecB^BB^, GDF9^GG^, and BMP15^AA^) and low-prolific (FecB^B+^, GDF9^AT^, and BMP15^BB^) Hu *sheep* were selected based on phenotype–genotype matching (Figure S1C–F). Growth traits, summarized in Table S4, revealed no significant differences in body height, body length, tube circumference, and rump width between high- and low-prolific Hu *sheep* (*p* > 0.05). However, high-prolific Hu *sheep* exhibited a significantly lower chest circumference than low-prolific Hu *sheep* (*p* < 0.05). Concentrations of LH, FSH, GnRH, and FS (Figure S2) showed significant differences between high- and low-prolific Hu *sheep* (*p* < 0.05), as detailed in Tables S5–S11. Post-slaughter analysis indicated no significant differences in initial body weight, carcass weight, slaughter rate, or organ weights, including pituitary, ovary, and uterus, between low- and high-prolific groups (*p* > 0.05) (Tables S12 and S13). Stroma and theca of developing follicles, as well as the granulosa and thecal-interstitial cells of large preovulatory and ovulating follicles, were identified through HE staining in Hu *sheep* with high and low prolificacy (Figure 1D).

### 3.2. Distinct Expression Profile of Granulosa Cells (GCs) and Theca Cells (TCs) Within the Dominant Follicles Were Identified in Sheep with Varying Prolificacies

**Figure 2:**
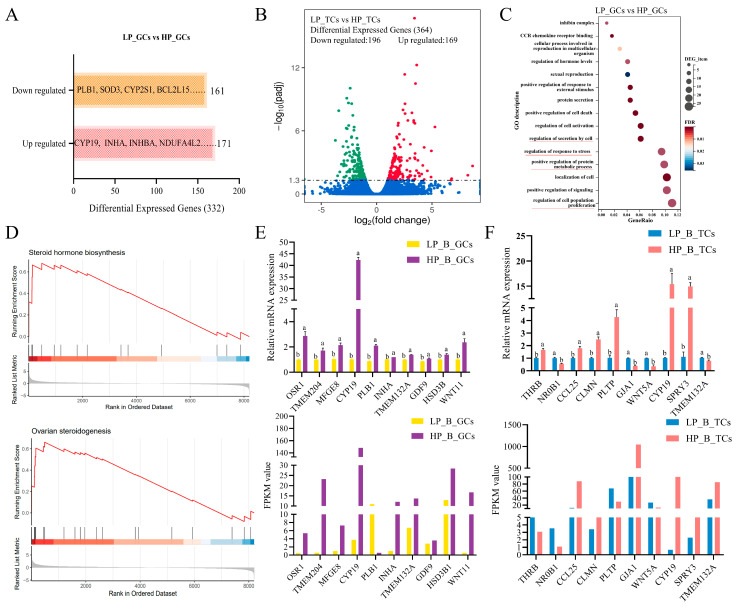
The transcriptional expression profile of dominant follicular cells was identified in *sheep* with varying prolificacies. Bar chart of up- and down-regulated DEGs in GCs (**A**) and Volcano plot of up- (red dots) and down-regulated (green) DEGs in TCs (**B**) from *sheep*’s dominant follicles with high and low prolificacy. The blue indicates no significant differences. (**C**) Significant GO terms of DEGs in *sheep* GCs. Red underlines highlight key GO terms related to our study. (**D**) GSEA analysis of Steroid Hormone Biosynthesis and Ovarian Steroidogenesis, two significant KEGG pathways in ovarian GCs. Validation of RNA-seq data accuracy by qRT-PCR.The gene expression was detected by qRT-PCR and normalized to ACTB, and the control set to one in *sheep* GCs (**E**) and TCs (**F**). Values without a common superscript (lowercase letter a or b) differ (*p* < 0.05, n = 3). The same as below.

To explore the mechanisms responsible for the selection of dominant follicles, RNA-seq analyses were performed on follicular granulosa cells (GCs) and theca cells (TCs) within the dominant follicles of Hu *sheep* with varying prolificacies. The detailed processing of sequenced reads and statistical analysis of raw data, along with mapping to the reference genome, are presented in Tables S14 and S15. A total of 332 DEGs were identified in GCs and 364 in TCs, meeting criteria of fold change > 1.5 and *p* < 0.05 (Figure 2A,B). GO enrichment and KEGG pathway analyses were conducted to investigate our biological relevance. In GCs, enriched biological processes included the regulation of secretion by cells, response to stress, protein metabolic process, and cell population proliferation (Figure 2C). KEGG pathways highlighted ovarian steroidogenesis and metabolism (Figure S3A). For TCs, enriched processes included hormone regulation and steroid biosynthesis, supported by pathways such as mTOR signaling and lysosomes (Figure S3B,C). Gene set enrichment analysis (GSEA) of GCs confirmed the enrichment of steroid hormone biosynthesis in *sheep* with a high prolificacy (Figure 2D). qRT-PCR validated the expression patterns of selected genes, confirming the reliability of our findings (Figure 2E,F). Principal component analysis (PCA) of RNA-seq peaks clearly distinguished TCs and GCs in Hu *sheep* with different prolificacies (Figure S3D). PC1 exhibited a significant separation between TC and GC samples. The identified common genes in ovarian follicular TCs and GCs of Hu *sheep* with varying prolificacies included INHBA, CDHR5, and CYP19 (Figure S3E). KEGG enrichment analysis of these common genes (HP_B_TCs versus. LP_B_TCs and HP_B_GCs versus. LP_B_GCs) highlighted pathways such as cytokine–cytokine receptor interaction, cortisol synthesis and secretion, NF-kappa B and MAPK signaling (Figure S3F).

### 3.3. The Metabolic Character Was Identified in the Dominant Follicular Fluid of Hu Sheep with Different Prolificacies

**Figure 3:**
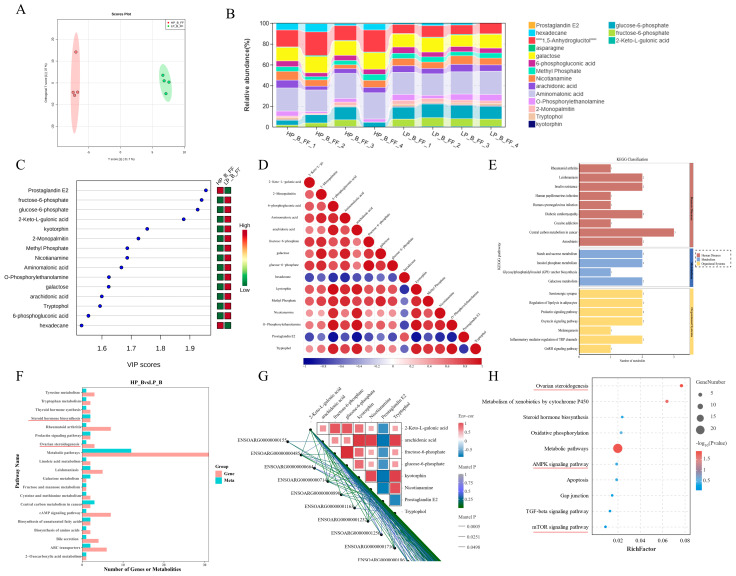
Dominant follicular fluid metabolomic profiling and integration with transcriptomic in ovarian cells of *sheep* with different prolificacies. (**A**) OPLS-DA score plot for the metabolomic datasets of dominant follicle fluid of Hu *sheep* with high and low prolificacy, denoted by high (HP_B_FF) and low prolificacy (LP_B_FF). (**B**) Heatmap shows the relative abundance of differential metabolites. (**C**) VIP-score plots of differential metabolites in dominant follicle fluid. (**D**) The correlation of differential metabolites. (**E**) KEGG classification analysis of differential follicle fluid metabolites. (**F**) Histogram of common KEGG pathways for the metabolomics (meta) and transcriptome (gene) of HP_B_GCs and LP_B_GCs, with different colors representing different omics. (**G**) Correlation map of the metabolomics (meta) and DE genes of GCs. (**H**) KEGG pathway analysis of genes correlated with NMN in GCs. Red underlines highlight key pathway related to our study.

After quality control of the OPLS-DA model, 287 metabolites were identified in follicular fluid samples from Hu *sheep* using GC-MS/MS. OPLS-DA analysis demonstrated a distinct classification of metabolites between high-prolific (HP_B_FF) and low-prolific (LP_B_FF) groups (Figure 3A). Twenty-five metabolites were found to be significantly differentially expressed in Hu *sheep* follicular fluid compared to the low prolificacy group, meeting criteria of *p*-value < 0.05, VIP >= 1, and fold change < 0.67 or >1.5, as illustrated in volcano plots (Figure 3B) and the VIP score among differential metabolites (Figure 3C). These metabolites belong to organic acid derivatives, lipids, organic oxides, and organic heterocyclic compounds. Correlation analysis revealed associations such as tryptophol and prostaglandin E2 (Figure 3D). Pathway enrichment analysis using KEGG indicated significant enrichment (*p* < 0.05) pathways, including prolactin signaling, oxytocin signaling, GnRH signaling, and central carbon metabolism in cancer (Figure 3E), distinguishing metabolic profiles between high and low prolificacy groups (Table S16). Notably, metabolites such as prostaglandin E2, sorbose, and fructose were significantly upregulated in the HP group, whereas myo-inositol, Fructose 2,6-biphosphate, Nicotinamide and several others were downregulated (*p* < 0.05) compared to the LP group. Integrated transcriptome and metabolome analysis revealed shared KEGG pathways, such as metabolic pathways, ovarian steroidogenesis, cAMP signaling, AMPK pathway, and amino acid biosynthesis between TCs (Figure S3G) and GCs (Figure 3F) in Hu *sheep* with high and low prolificacy. Genes associated with NMN in TCs also exhibited positive and negative correlations (Figure S3H). Correlation analysis showed that NMN abundance in follicular fluid positively correlated with specific differential genes in GCs (TRERF1, EYA2, CD300E, etc.) and negatively correlated with others (LOC101104500, SEMA6A, etc.) (Figure 3G). KEGG analysis indicated that NMN activated metabolic pathways, steroid biosynthesis, oxidative phosphorylation, as well as AMPK/mTOR pathways in GCs (Figure 3H).

### 3.4. NMN Alleviates Cell Proliferation and Apoptosis in LPS-Induced Granulosa Cells

**Figure 4:**
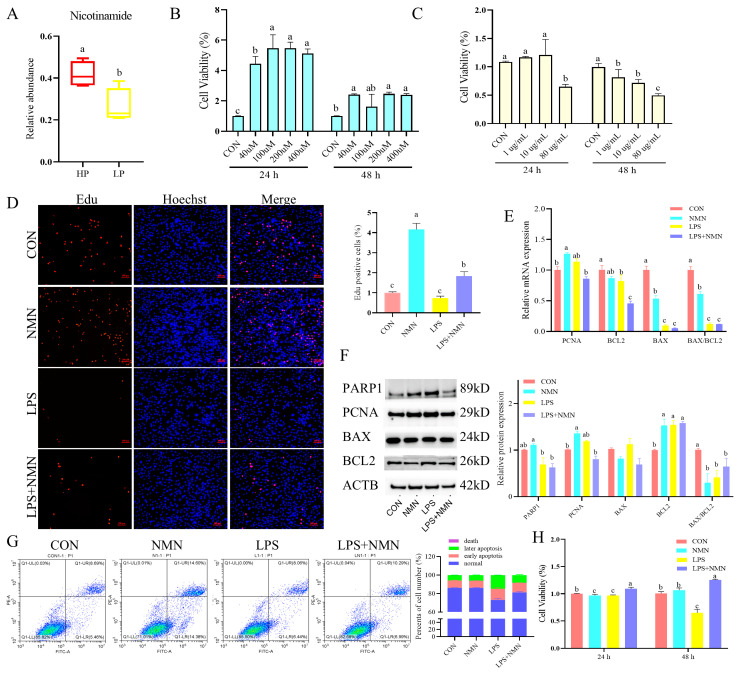
NMN alleviates LPS-induced granulosa cell proliferation and apoptosis. (**A**) The relative abundance of nicotinamide in the follicular fluid of Hu *sheep* with different prolificacies. (**B**) The suitable concentration of NMN was screened in Hu *sheep* GCs by CCK-8 assay. (**C**) The suitable concentration of LPS was screened in Hu *sheep* GCs by CCK-8 assay. (**D**) Analysis of Edu-positive cells in different groups. Scale bar in figures = 100 μm. (**E**) qPCR analysis of proliferation and apoptosis-related genes in treated GCs. (**F**) The protein expression of proliferation and apoptosis-related genes in treated GCs. (**G**) Assessment of apoptosis using Annexin V-FITC/PI and flow cytometry in treated GCs. Numbers adjacent to the outlined areas indicate the percentage of the gated population in each group. (**H**) Cell viability of *sheep* GCs in four experimental groups by CCK8-based assay. a–c: Values without a common superscript differ (*p* < 0.05, n = 3).

Nicotinamide was upregulated (*p* < 0.05) in the HP group compared to the LP group (Figure 4A). To investigate the effects of NMN on LPS-induced injury in Hu *sheep* GCs, cells were isolated and cultured in vitro. Immunofluorescence microscopy confirmed the expression of FSHR and LHR on cell membranes, demonstrating GC purity of up to 90% (Figure S4). Cell viability was assessed using CCK-8, showing that 100 μM NMN notably enhanced cell viability 5-fold compared with the control group; while the cells were exposed to 80 μg/mL of LPS, the survival rate of the cells decreased by about 50% compared with the control group (Figure 4B,C), establishing optimal conditions for subsequent experiments. Further analysis revealed that NMN promoted cell proliferation 4.16-fold via the Edu positive cell ratio (*p* < 0.05) and reduced the cell apoptosis by 7.9%, as evidenced by Edu assay and flow cytometry (Figure 4E–G). NMN supplementation also decreased mRNA expression of *BAX* and the *BAX/BCL2* ratio (*p* < 0.05), while increasing *PCNA* expression (*p* < 0.05) (Figure 4E). Moreover, NMN mitigated apoptosis induced by LPS, evidenced by a decreased BCL2 protein expression (*p* < 0.05), with no significant changes in PARP1 levels (*p* > 0.05) (Figure 4F).

### 3.5. NMN Impairs LPS-Induced Granulosa Cell Mitochondrial Dysfunction and Attenuates Oxidative Stress

**Figure 5:**
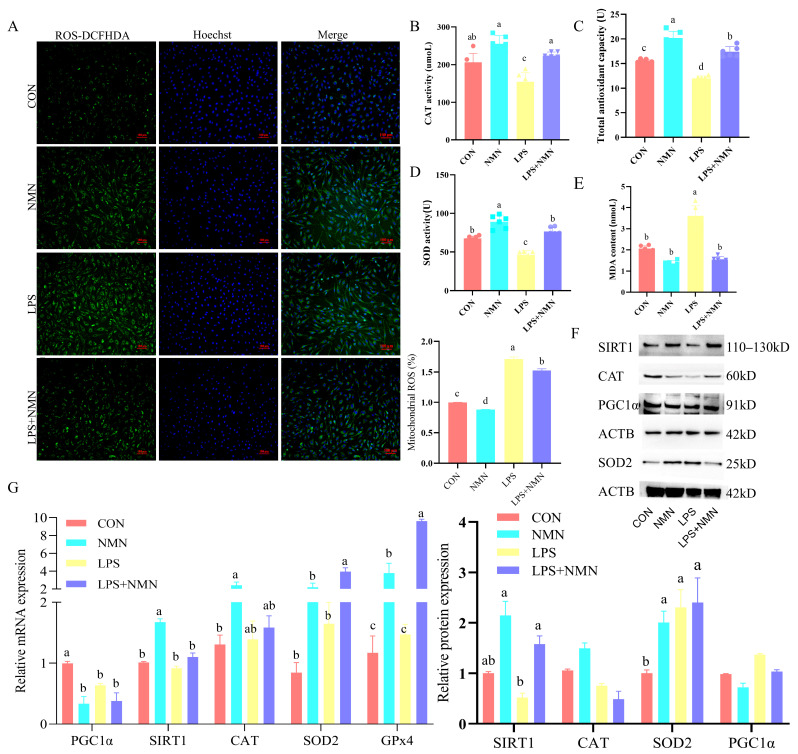
NMN attenuates LPS-induced granulosa cell oxidative stress. (**A**) ROS content in treated GCs by ROS-DCFHDA probe. CAT activity (**B**), total antioxidant capacity (**C**), SOD activity (**D**), and MDA content (**E**) in treated GCs detected by ELISA. (**F**) Protein levels of SIRT1, CAT, SOD2, and PGC1α in GCs after four treatments by Western blot. (**G**) mRNA levels of oxidative stress-associated genes in treated GCs by qPCR. a–d: Values without a common superscript differ (*p* < 0.05, n = 3).

Mitochondrial ROS accumulation was mitigated by 88% after NMN supplementation in LPS-induced GCs (*p* < 0.05; Figure 5A). To assess the antioxidative effects of NMN in LPS-induced GCs, the results showed that the MDA levels significantly decreased to 42% in LPS-NMN-co-treated GCs (*p* < 0.05; Figure 5E). NMN supplementation significantly increased the total antioxidant capacity 1.3-fold, CAT 1.3-fold, and SOD activity 1.1-fold (*p* < 0.05; Figure 5B–D). Western blot analysis showed an increased protein expression of SIRT1 and SOD2 (*p* < 0.05; Figure 5F), along with decreased PGC1α mRNA levels (Figure 5G). In the LPS+NMN groups, NMN supplementation significantly increased the total antioxidant capacity 1.5-fold, CAT 1.4-fold, and SOD activity 1.6-fold (*p* < 0.05; Figure 5B–D), accompanied with higher SOD2, GPx4 transcripts, and SIRT1 protein expression (*p* < 0.05; Figure 5G) compared to the LPS-only group, suggesting a protective effect against LPS-induced damage.

**Figure 6:**
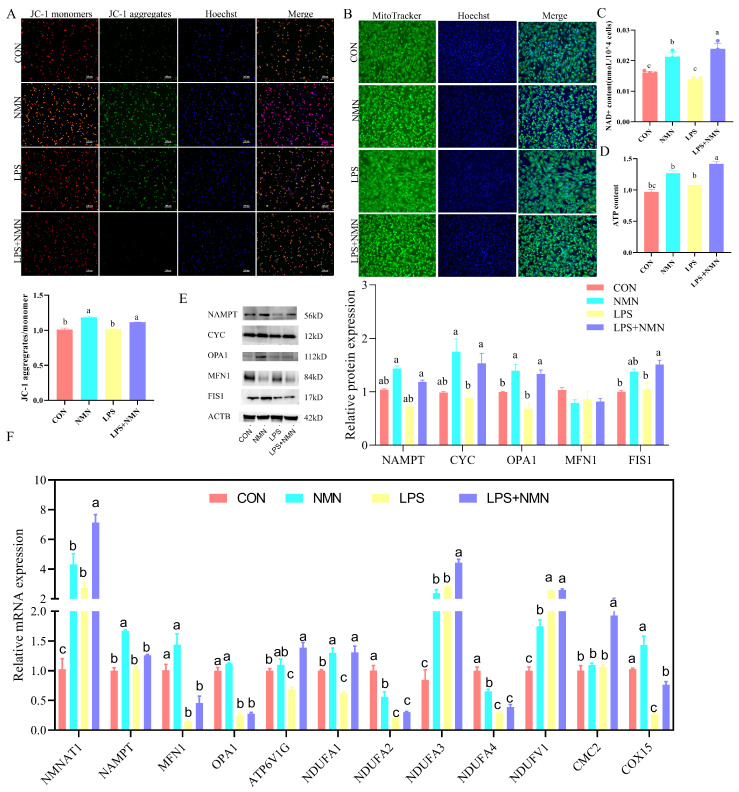
NMN prevented LPS-induced granulosa cell mitochondrial dysfunction. (**A**) Mitochondrial membrane potential in treated GCs detected by JC-1 assay. (**B**) Localization of mitochondria in treated GCs. (**C**) NAD+ content and (**D**) ATP content was detected by ELISA. (**E**) Protein levels of NAMPT, CYTC, OPA1, MFN1, and FIS1 in treated GCs by Western blot. (**F**) mRNA expression of ATP synthase, NADH dehydrogenase, Cytochrome C oxidase, NMNAT1, NAMPT, MFN1, and OPN in treated GCs by qPCR. a–c: values without a common superscript differ (*p* < 0.05, n = 3).

Further exploration of mitochondrial dynamics revealed an increased mitochondrial membrane potential in LPS-NMN-co-treated GCs (*p* < 0.05; Figure 6A), coupled with an altered mitochondria morphology in NMN-treated GCs (Figure 6B). Moreover, NMN supplementation significantly improved the lower ATP and NAD content in LPS-stimulated GCs (*p* < 0.05; Figure 6C,D), correlating with dynamic protein levels of NAPMT, CYC, OPA1, and FIS1 (*p* < 0.05; Figure 6E). Accordingly, qPCR analysis showed that NMNAT1, NAMPT, the mitochondrial fission and fusion genes (MFN1, OPA1), ATP synthase (ATP6V1G), NADH dehydrogenase (NDUFA1, NDUFA2, NDUFA3, NDUFA4, NDUFV1), and Cytochrome C oxidase (CMC2, COX15) were differentially altered upon LPS-stimulated GCs relative to control groups. However, NMN treatment increased the expression of these genes (Figure 6E).

### 3.6. NMN Improved Steroidogenesis and Ovarian Responsiveness in LPS-Induced GCs

**Figure 7:**
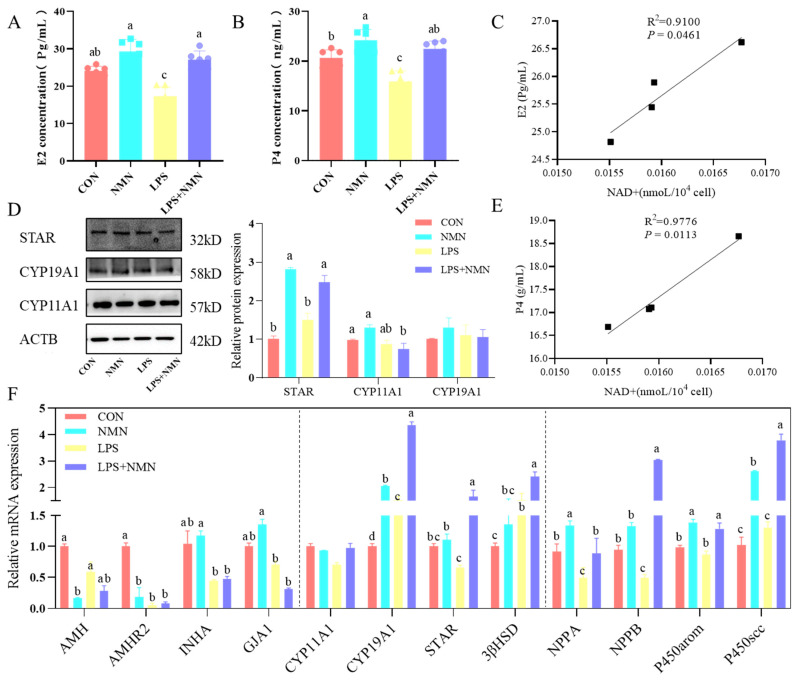
NMN enhanced LPS-induced granulosa cell steroidogenesis. (**A**,**B**) E2 and P4 levels in GC culture medium were detected by ELISA. (**C**) Correlation between NAD content and E2 concentration in cultured GCs. (**D**) Western blot detected the protein levels of steroid enzymes, including STAR, CYP11A1, CYP19A1, in treated GCs. (**E**) Correlation between NAD content and P4 concentration in GCs. (**F**) qPCR determined the mRNA levels of genes involved in follicular development (INHA, GJA1), ovarian responsiveness (AMH, AMHR), steroid enzymes (CYP11A1, CYP19A1, STAR, 3β-HSD), natriuretic peptide precursor A and B (NPPA, NPPB), and P450arom, P450scc in treated GCs. a–d: Values without a common superscript differ (*p* < 0.05, n = 3).

To investigate the impact of NMN on steroidogenesis in LPS-induced GCs, steroid hormone levels and associated genes were assessed using ELISA and qPCR. NMN supplementation significantly restored E2 and P4 concentrations (*p* < 0.05) in LPS-induced GCs (Figure 7A,B), accompanied by an increased expression of CYP11A1 and STAR proteins (Figure 7D), and elevated mRNA levels of CYP19A1, STAR, 3βHSD (Figure 7F). The relative mRNA expression of CYP11A1 showed no significant differences (*p* > 0.05) among the groups (Figure 7F). In addition, the expressions of natriuretic peptide precursor A and B (NPPA, NPPB) were decreased by LPS stimulation and improved by NMN treatment, along with the genes involved in steroid synthesis (P450arom and P450scc) in GCs (Figure 7F). Further, NAD content was positively correlated (*p* < 0.05) with the E2 and P4 concentration (Figure 7C,E) in GCs. Additionally, NMN supplementation significantly increased (*p* < 0.05) the expression levels of follicular development-associated genes, including INHA and GJA1, and decreased the transcripts of ovarian responsiveness markers (AMH and AMHR2) in GCs (Figure 7F) compared to those in the control group.

### 3.7. NMN Impaired the AMPK/mTOR Pathway in LPS-Induced GCs

**Figure 8:**
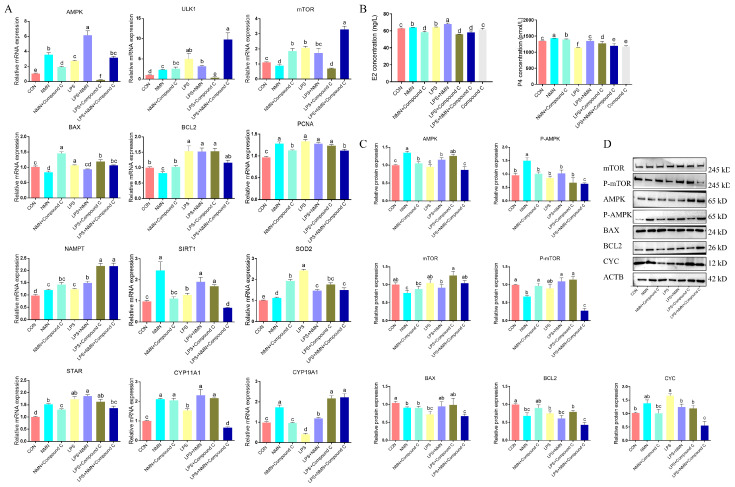
NMN disrupted AMPK signaling pathway in LPS-induced granulosa cells. (**A**) qPCR determined the mRNA levels of associated genes in different treated GCs. (**B**) E2 and P4 concentrations in treated GCs. (**C**,**D**) Western blot detected the protein expression in treated GCs. a–f: Values without a common superscript differ (*p* < 0.05, n = 3).

Mechanistically, NMN’s effects on AMPK/mTOR signaling in LPS-induced GCs were examined (Figure 8C,D). NMN supplementation significantly enhanced AMPK and ULK1 expression (*p* < 0.05), while decreasing mTOR (*p* < 0.05) (Figure 8A). Western blot analysis revealed that NMN supplementation decreased the phosphorylation of AMPK (*p* < 0.05) alongside a lower mTOR phosphorylation (*p* < 0.05) in LPS-induced GCs. To further determine whether AMPK/mTOR signaling activation was associated with NMN-regulated steroidogenesis, we examined the effect of AMPK inhibitor Compound C on markers of cell apoptosis and steroid hormone biosynthesis. As shown in Figure 8, Compound C treatment decreased the expression of AMPK and phosphorylated AMPK (*p* < 0.05) and enhanced E2 concentration (*p* < 0.05) in GCs pretreated with LPS and NMN (Figure 8A,B). In addition, AMPK inhibitor Compound C also significantly promoted the expression of mTOR and phosphorylated mTOR expression as well as cell apoptosis-associated markers (BAX, BCL2), while inhibiting steroid hormone enzymes (STAR and CYP11A1), and it regulated redox markers (NAMPT, SIRT1, SOD2, CYC) (Figure 8A,D). These results indicate that NMN mediation of AMPK/mTOR activation is important for *sheep* granulosa cell steroidogenesis.

## 4. Discussion

To elucidate whether any molecules in FF may signal ovulation within dominant follicles, we screened FF differential metabolites in the Hu *sheep* with high and low prolificacy, and further explored their effects and mechanisms in GCs by integrating metabolic and RNA-seq analysis. Our study revealed that Nicotinamide is highly expressed in the follicular fluid of dominant follicles associated with high prolificacy in *sheep*. Further experiments demonstrated the role and regulated mechanism of NMN in *sheep* LPS-induced GCs (Graph abstract). These findings propose that NMN might serve as a novel metabolic marker for dominant follicle development, offering a theoretical foundation for its potential application in reproduction.

The metabolic state significantly influences reproduction [3], and furthermore, a well-regulated metabolism is essential for follicular development. A study by Lin et al. highlighted that the accumulation of toxic metabolites within follicular fluid and ovarian stroma adversely affects ovarian function [50], implicating metabolic dysfunction, including inflammation and aging, that can impair cellular or organismal health. The establishment of dominant ovarian follicles in *sheep* involves complex and dynamic processes, with the rapid proliferation of GCs driving follicular maturation and female fertility [51]. Despite the known importance of dominant follicle selection, the precise mechanisms linking prolificacy to follicular dynamics remain poorly understood. Our metabolomic analysis of ovarian dominant follicle fluid revealed a higher abundance of nicotinamide in Hu *sheep* with HP relative to LP, highlighting its positive correlation with prolificacy. A limitation of this study is the small number of animals in the metabolomic analysis, primarily due to sample availability and strict inclusion criteria. While the findings offer valuable insights into the metabolic profile of Hu *sheep*, larger studies are needed to validate these results and improve the generalizability of our conclusions. The structure of a follicle comprises multiple layers, including the outer TCs [52], which produce androgens, and a thicker layer of GCs. We found a notable correlation between the abundance of nicotinamide, a crucial follicular metabolite, and the transcriptomic expression of GCs, identifying several genes—including PLB1, SGMS2, ACSBG1, and CYP19—associated with hormone secretion, nicotinamide metabolism and the AMPK signaling pathway. These findings underscore the intricate interplay between metabolic factors and follicular health, further elucidating the relationship between metabolic balance, including cellular metabolism and reproductive performance.

The impact of β-Nicotinamide mononucleotide, a metabolite derived from nicotinamide present in Hu *sheep* FF, on GC functions was the central focus of our study. Previous studies have shown that nicotinamide is present in human FF [22], but its role in *sheep* prolificacy, especially in Hu *sheep*, is less explored. Our study fills this gap by demonstrating higher nicotinamide levels in the FF of dominant follicles in high prolificacy Hu *sheep* compared to low prolificacy *sheep*. This difference may be influenced by genetic and biochemical factors, as previous research links higher nicotinamide levels in FF to larger follicle development and improved oocyte quality [22]. We used markers such as FecB, GDF9, and BMP15 to classify prolificacy and investigate their relationship with nicotinamide. Genetic differences in nicotinamide metabolism, such as variations in NAMPT activity [25], may account for the increased nicotinamide concentrations in prolific *sheep*. However, further studies are needed to explore the genetic and molecular mechanisms behind this observation. Additionally, comparisons with other *sheep* breeds or species would help determine whether this pattern is unique to Hu *sheep* or more broadly applicable. Nicotinamide, an essential compound in the body, serves as a precursor to NMN, a critical intermediate in NAD+ biosynthesis [53]. Previous studies showed that NMN was able to increase the NAD concentration in HepG2 cells for 48 h [54], suggesting its stable ability in media over longer treatments. Emerging evidence indicates that NMN treatment enhances NAD+ levels [55] and promotes SIRT1 activation [56]. This activation is linked to improvements in mitochondrial rejuvenation and anti-apoptotic pathways [25]. Supplementation with NMN in mice has been shown to protect against reproductive abnormalities induced by zearalenone by enhancing NAD+ levels [57]. Our findings support these observations, demonstrating that NMN supplementation treatment significantly elevates both NAD+ and SIRT1 levels in GCs, alongside increased protein expression of NAPMT and CYTC. Importantly, our results reveal that NMN treatment increases mitochondrial membrane potential in both NMN-treated GCs and those co-treated with LPS and NMN. Additionally, NMN downregulated mitochondrial fission proteins (Fis1) while upregulating fusion proteins (Mfn1 and Mfn2), corroborating previous findings [58]. Previous studies have highlighted that mitochondrial stress in GCs can impair the maturation of the cumulus–oocyte complex, promote follicle apoptosis, disrupt metabolic processes, and contribute to ovarian fibrosis [59]. Integrating our findings with recent research on mitochondria-targeted interventions, we propose that mitochondrial rejuvenation is a key mechanism underlying the restoration of steroidogenesis and promotion of follicle development. Therefore, NMN may ameliorate LPS-induced GC dysfunction in *sheep*, at least partially, by enhancing mitochondrial fusion and fission dynamics.

Ovarian dysfunction can lead to infertility through premature follicle depletion and the unregulated proliferation of GCs [60]. Recent studies have demonstrated that NMN improves cell viability and reduces apoptosis in LPS-treated MH-S cells [27]. Consistent with these findings, our research indicates that NMN enhances both proliferation and viability in LPS-treated GCs. It appears to exert protective effects by scavenging free radicals [61] and decreasing lipid peroxidation in GC membranes [62]. Indeed, our results show that malondialdehyde (MDA), a marker of lipid peroxidation, was significantly elevated in LPS-treated cells. Furthermore, we observed that LPS treatment increased ROS levels while decreasing the activity of antioxidant enzymes, including total antioxidant capacity (T-AOC), catalase (CAT), and superoxide dismutase (SOD) in *sheep* GCs. Notably, NMN reversed these detrimental effects. Previous research has established that replenishing NAD+ with NMN effectively alleviates oxidative stress and apoptosis induced by LPS, particularly through its impact on SIRT1 expression [63]. Moreover, the NAD+/SIRT1 pathway is often inhibited during heightened inflammatory states [38]. Our findings align with this literature, emphasizing the importance of restoring mitochondrial function as a mechanism through which NMN mediates improvements in LPS-induced injury via enhanced SIRT1 gene expression.

Our findings support the notion that NMN effectively enhances NAD+ biosynthesis [64] and mitigates oxidative stress [65]. Similar protective effects have been observed in aged blood vessels [66,67], ram sperm [61], and ocular oxidative stress models [68]. Notably, we demonstrated that NMN is crucial for attenuating LPS-induced excessive reactive oxygen species (ROS) production, reducing antioxidant capacity, and elevating circulating oxidative stress biomarkers in GCs. Intracrine NAD-dependent circadian steroidogenic activity has been documented in various tissues [69]. In our study, NMN alleviated decreases in estradiol (E2) and progesterone (P4) levels in LPS-treated GCs and influenced the expression of steroidogenic enzymes, such as the steroidogenic acute regulatory (STAR) protein, as well as CYP19A1 and P450scc mRNA. The up-regulation of CYP19A1 expression promotes 17β-estradiol synthesis, which is known to inhibit apoptosis during follicle development [70]. These findings suggest that NMN enhances steroid hormone levels and the expression of enzymes necessary for steroidogenesis. We speculate that granulosa cells isolated from follicular fluid, when cultured in serum-supplemented medium, may exhibit a tendency toward luteinization. This process could enhance their capacity for androgen synthesis, which in turn supports estradiol production [71]. Further studies are needed to fully understand the regulatory mechanisms of steroidogenesis. The ovarian responsiveness marker anti-Müllerian hormone levels (AMH) refers to the ability of the ovaries to respond to hormonal stimuli [66]. Our findings indicate that NMN significantly influences candidate genes associated with follicle development and ovarian responsiveness markers, such as INHA and AMH, suggesting that NMN may impact ovarian responsiveness in *sheep* by modulating the expression of marker genes. NMN is also shown to enhance mitochondrial energy metabolism, reduce cell apoptosis, and alleviate oxidative stress by activating the SIRT1-PGC1α [32] and SIRT1/NAD+ pathways [38]. This aligns with our findings, which indicate that NMN’s protective effects in LPS-treated GCs involve reduced oxidative stress, decreased cell apoptosis, and restored mitochondrial function through the activation of the AMPK/mTOR signaling pathway. Additionally, NMN has been reported to attenuate airway epithelial barrier dysfunction by inhibiting SIRT3 SUMOylation in asthma models [72]. In the context of follicular metabolism, AMPK may further mitigate ROS toxicity by enhancing antioxidant enzyme activity [73]. Consistent with the findings of Hopp et al., who demonstrated that NMN inhibits hepatocellular carcinoma progression by inducing autophagy and ferroptosis through AMPK signaling [34], our results underscore the multifaceted protective roles of NMN in granulosa cells.

Ovarian infection and inflammation are significant contributors to low prolificacy in livestock [35], yet the mechanisms through which inflammation impacts reproductive performance remain poorly understood. Specifically, LPS stimulation led to substantial differences in oxidative stress, mitochondrial function, ovarian steroidogenesis, and AMPK/mTOR hyperactivation between control and LPS-treated GCs, most of which were ameliorated by NMN supplementation. Previous reports have highlighted NMN’s potential in preventing and protecting against various ocular diseases [68], and NMN supplementation has been shown to protect oocytes from age-related deterioration [31]. Our research suggests that NMN could serve as a key biomarker of prolificacy. In fertility, NMN is likely more linked to improving ovarian health in compromised conditions. Furthermore, the role of nicotinamide as a precursor to NMN underscores its importance in maintaining cellular health and promoting physiological homeostasis. This relationship highlights the need for a deeper understanding of the complex interplay between these molecules and their implications for human health and well-being. Further research involving ovarian tissues, as well as co-cultures of granulosa and theca cells, and whole dominant follicles may provide deeper insights into NMN’s effects [74]. Disruptions in the metabolic balance of GCs can adversely affect the microenvironment of follicles, ultimately impacting oocyte maturation and developmental potential.

## 5. Conclusions

In conclusion, this study provides valuable insights into the metabolites regulating the selection of dominant follicles for ovulation associated with prolificacy, and it highlights that NMN protected LPS-induced GCs against cell apoptosis, oxidative stress, mitochondrial dysfunctions, and impaired ovarian steroidogenesis via the AMPK/mTOR pathway. Addressing the acknowledged limitations and pursuing future research directions will advance our understanding and application of NMN as a potential novel metabolic biomarker in enhancing ovarian function.

## Data Availability

The data that support the findings of this study are available from the corresponding author upon reasonable request.

## Supplementary Materials

The following supporting information can be downloaded at: https://www.mdpi.com/article/10.3390/antiox14010034/s1, Figure S1: Polymorphism analysis of BMPR-IB, GDF9 and BMP15 genes in *sheep* with different prolificacies. The number of tracks differ on the different migration pictures. Figure S2: Blood biochemical of *sheep* with different prolificacies. Figure S3: Integrated analysis of metabolome and transcriptome. Figure S4: Verification of the integrity of *sheep* follicular granulosa cells. Table S1: Details of PCR primers. Table S2: Primer sequences and predicted product size of genes used for reverse transcription and quantitative real-time PCR. Table S3: Details of specific antibody used for Western blot in the experiment. Table S4: Analysis of birth weight and body size index of *sheep* with high and low prolificacy. Table S5: Blood hormone concentration during estrus cycle of *sheep* with high and low prolificacy. Blood LH (Table S6), FSH (Table S7), GnRH (Table S8), FS (Table S9), E2 (Table S10) and P4 (Table S11) concentration within 3 h after estrus of *sheep* with high and low prolificacy. Table S12: Slaughter traits of *sheep* with high and low prolificacy. Table S13: Organ weight and its index of *sheep* with high and low prolificacy. Table S14: Alignment of sequenced reads processing with statistics of raw data in *sheep* ovarian cells with high and low prolificacy. Table S15: Alignment of the clean reads mapping with the reference genome in *sheep* ovarian cells with high and low prolificacy. Table S16: Significantly enriched KEGG pathway by differential metabolites.
