# Supplementary material for: β-Nicotinamide Mononucleotide Reduces Oxidative Stress and Improves Steroidogenesis in Granulosa Cells Associated with Sheep Prolificacy via Activating AMPK Pathway
Source: Antioxidants (Basel). 2024 Dec 30;14(1):34. doi: 10.3390/antiox14010034 (PMC11762531; doi:10.3390/antiox14010034)
Supplement: Supplementary file 1 [file antioxidants-14-00034-s001.zip › antioxidants-3335521-supplementary.pdf]

Supplementary figures and tables

Figure S1. Polymorphism analysis of *BMPR-IB*, *GDF9* and *BMP15* genes in sheep with different prolificacy

(A) Whole blood genome DNA bands of experimental sheep by agarose gel. (B) Verification of the PCR products size of *FecB*, *GDF9*, and *BMP15* by agarose gel. (C) The mutation of *BMPR-IB* in Hu sheep was detected by PCR-SSCP. (D) The mutation of *BMP15* in Hu sheep was detected by PCR-SSCP. (E) The mutation of *GDF9* in Hu sheep was detected by PCR-SSCP. (F) The mutation of *BMPR-IB* in Hu sheep was detected by PCR- RFLP. (G) The whole estrus cycle of Hu sheep was identified by Giemsa method.

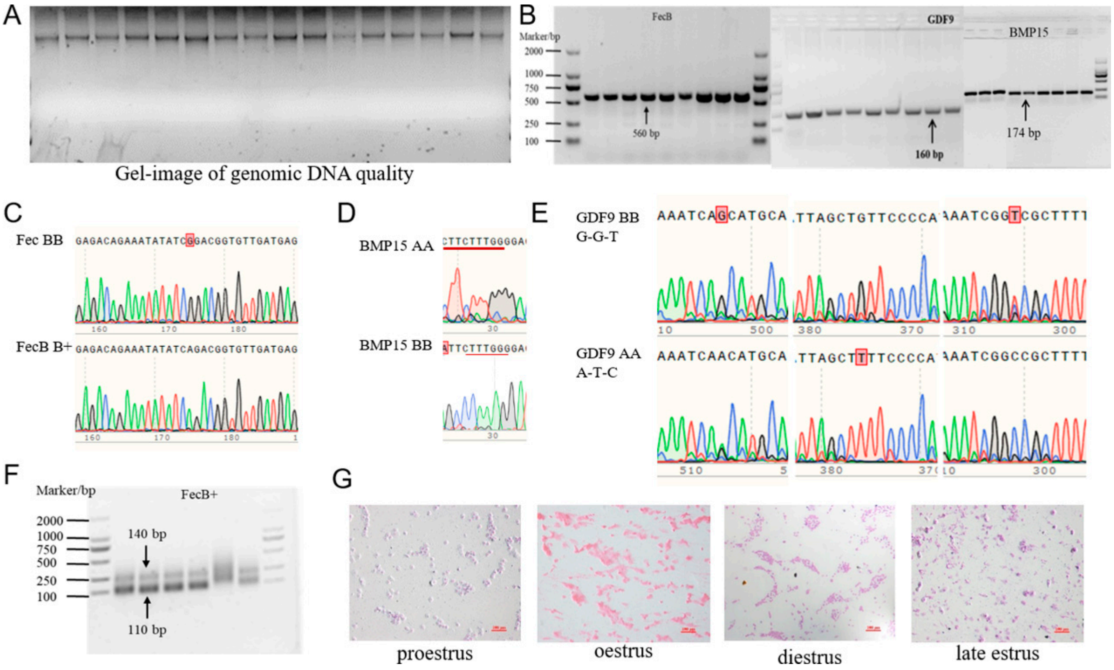

Figure S2. Blood biochemical of sheep with different prolificacy

The concentration of LH (A), GnRH (B), FSH (C), FS (D) in the blood of Hu sheep during the estrus cycle and 2.5 h before slaughter. a–b Values without a common superscript differ ($P < 0.05$).

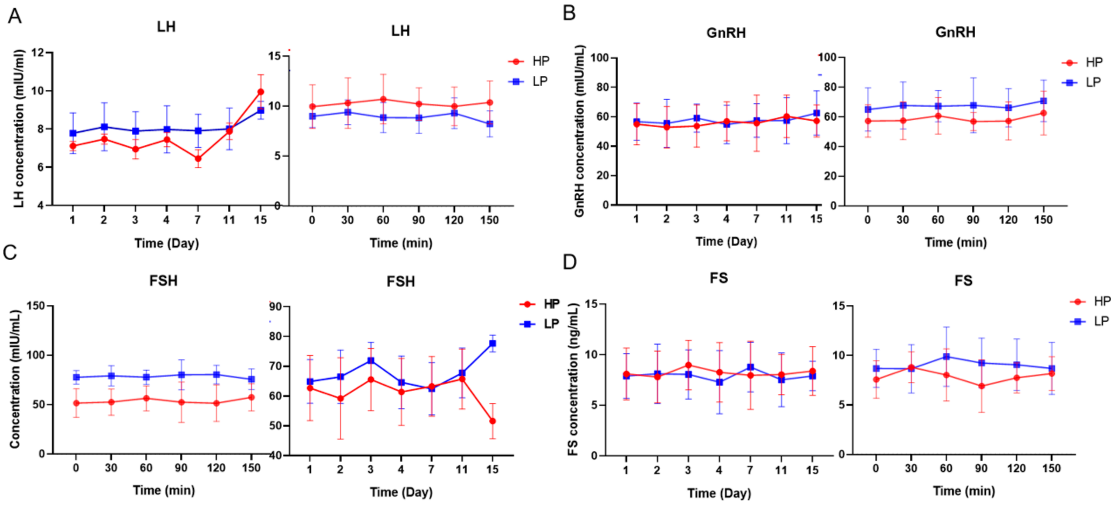

Figure S3. Integrated analysis of metabolome and transcriptome

Significantly enriched KEGG pathway by DEGs of GCs (A) and TCs (B). Significant GO terms by DEGs of GCs (C). (D) PCA plot for transcriptomic datasets of HP_B_GCs, LP_B_GCs, HP_B_TCs and LP_B_TCs. (E) The common genes of integrated RNA-seq with HP_B_GCs, LP_B_GCs, HP_B_TCs

and LP_B_TCs. (F) Common KEGG pathway of four groups by integrated RNA-seq analysis. (G) The histogram of the common KEGG pathways with the metabolomics (meta) and the transcriptome (gene) of HP_B_TCs and LP_B_TCs, different colors represent different omics. (H) The correlation heatmap of the metabolomics (meta) and the transcriptome (gene) of HP_B_TCs. and LP_B_TCs. (I) KEGG pathway analysis of genes in correlation with NMN.

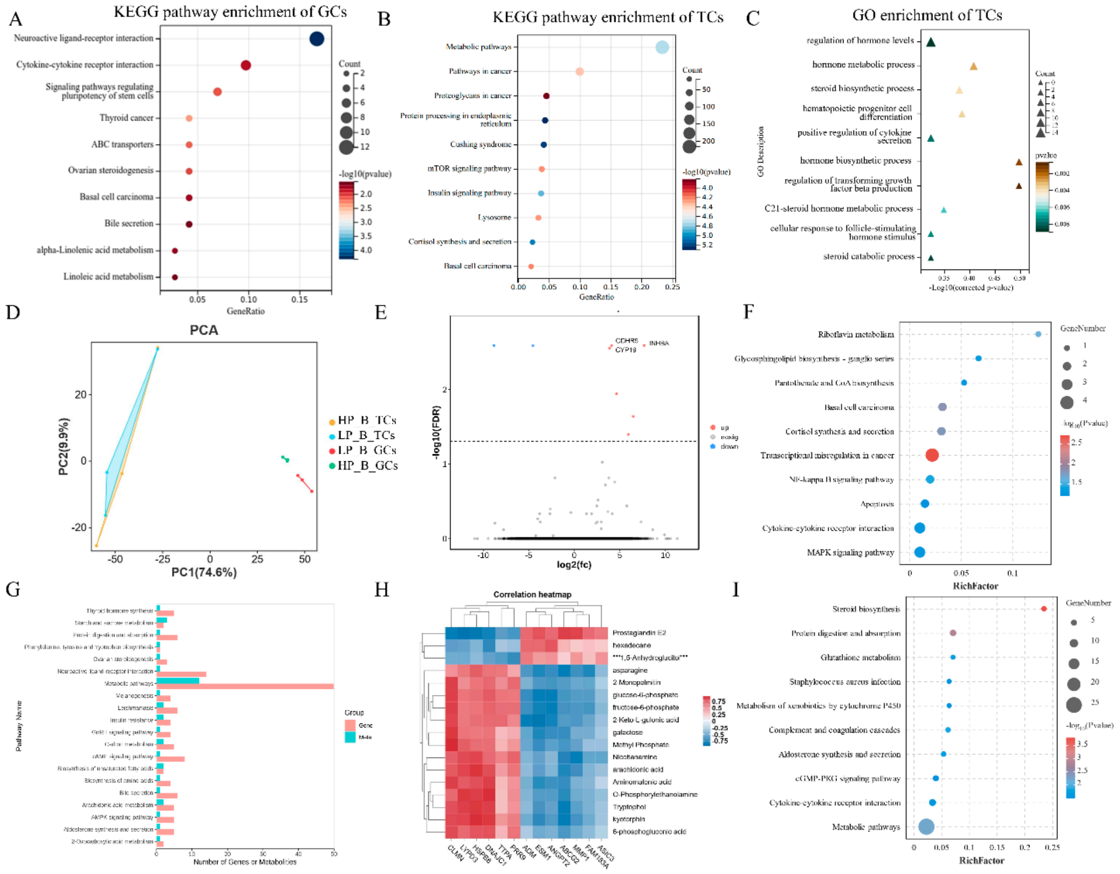

Figure S4. Verification the integrity of sheep follicular granulosa cells

(A) The morphological features of Hu sheep follicular GCs *in vitro*. (B) Identification of GCs by specific markers; (C-D) Growth characteristics of GCs between different generations. Scale bar in figures=100 μm.

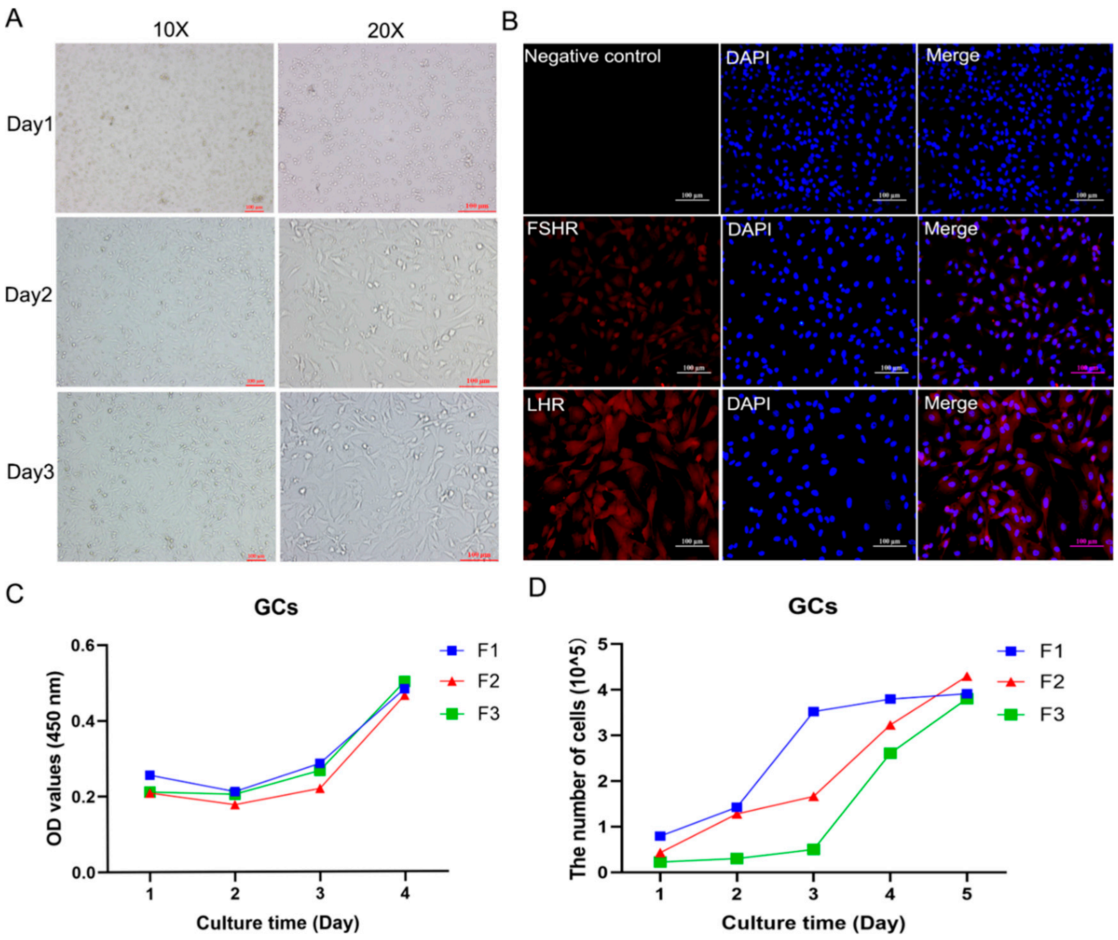

Supplementary tables

Table S1 Details of PCR primers

Gene	GenBank No.	Primer sequences (5′-3′)	Product length (bp)	Usage
FecB	NC_019463.1	F: GTCGCTATGGGGAAGTTTGGATG<br>R: CAAGATGTTTTCATGCCTCATCAACACGGTC	140	PCR-RFLP
	NW_011942424.1	F: GTGCCGTGAACGCACTAACA<br>R: AGACAAAAACGTGCTCCTTCAA	560	PCR-SSCP
BMP15	NM_001114767.2	F: GTTCTTGAGTTCTGGTGG<br>R: AATACTGCCTGCTTGACG	174	PCR-SSCP
GDF9	-	F: ACCCAAATAAAGGCACCA<br>R: CAAAGCAGCAAAACCAAA	768	PCR-SSCP
		F: TTCGTCGGAGCCTCAGGA<br>R: TGCTGATTTGGAGGGTGA	1215	PCR-SSCP

	F: gtgtgtaggagcagattggttaatgg	1123	PCR-SSCP
	R: ggttcctgagaagaaaaacaatccaacag		
	F: AAATAGCAGTTCTCTGCTCTCTGGAAT	600	PCR-SSCP
	R: GGCTTGGAAGAATTAGCAAGGAACAC		

Table S2 Primer sequences, predicted product size of genes used for reverse transcription and quantitative real-time PCR

Gene	Primer sequence (5′- 3′)	Product size (bp)	Accession number
ACTB	F-TCAGCAAGCAGGAGTACGAC<br>R-ACGAGGCCAATCTCATCTCG	138	NM_001009784.3
GPx4	F-ACATTGAAACCCTGCTGTCC<br>R-TCATGAGGAGCTGTGGTCTG	216	XM_005695962.3
SOD2	F-GTGAACAACCTCAACGTCGC<br>R-GCGTCCCTGCTCCTTATTGA	300	XM_018053428.1
CAT	F-CACTCAGGTGCGGGATTTCT<br>R-ATGCGGGAGCCATACAGG	159	XM_005690077.3
PCNA	F-AGTGGCGTGAACCTACAGAG<br>R-GCCAAGGTGTCCGCATTATC	457	XM_005688167.3
OSR1	F-GAGAAGTTCACCACCCCCTG<br>R-CTGGTGCTGGCAAGGTTTTG	271	XM_004005704.4
TMEM204	F-ATGCTACCTGAACATCGGCG<br>R-GCTGATGCCATGTGGTCAAG	300	XM_027961769.3
MFGE8	F-AGTGGAAGCTCGTGGGAATG<br>R-GTGATGCAGCGGTAGTGGTA	286	XM_027957063.2
PLB1	F-GTCCAAGGGGCAAACATCAAC<br>R-TGCCTCTCAAACAGGGAACAA	203	XM_042245933.2
INHA	F-AGGAGGGCCTCTTCACGTAT<br>R-GCTATTGGTGGCAGTGGTCT	122	NM_001308579.1
TMEM132A	F-GCGTGGAGTTTGTGACGTTT<br>R-GCACCAGAAGATGAACGGGA	258	XM_027959796.2
GDF9	F-GGCGGTCGGACATCGGTATG<br>R-GGATGGTCTTGGCACTGAGGAG	91	NM_001142888.2
WNT11	F-GGGTGAGGAGCAGAACTCG<br>R-CGAGCTGCCTGGAAAGGA	127	XM_060399452.1
THRB	F-CAAATGAACCAGAGCCCGAC<br>R-TGGGATCTGTCTTCTTCCGC	151	NM_001190391.1
NR0B1	F-TCCCAAGATCTCCCTGCTGA<br>R-CACAGCCAGATGGGGTAGAC	159	XM_027963039.2
CCL25	F-TGTCTCGCGCCTGTTTATCC<br>R-CATGCCCAATTTACTTTTCGGTCC	212	XM_042249414.2
CLMN	F-TTAACCAGGAAGGCCAACCAC<br>R-AGAGCAGCAGGCAGTAAACC	148	XM_027957369.3
PLTP	F-ATTCCTCTCCACGTTCATCACCTC<br>R-GCAGCGAATTGAGAAGCACAGTC	101	XM_060397065.1
GJA1	F-TGCAAAAGAGATCCCTGCCC<br>R-GAGACACCAACGACACGACA	103	XM_004011159.5

WNT5A	F-TTAATTCCGGCTCCACTTGTTG<br>R-CCTGGGCGAAGGAGAGAAAT	141	XM_042236058.1
CYP19/CYP19A1	F-GGCATGCATGAGAAAGGCAT<br>R-GAGGGTCAACACGTCCACAT	195	NM_001123000.1
SPRY3	F-CCAGTCCATCATCCGAACCC<br>R-GCGCAGTTGTCTTCATCGTC	296	XM_004022248.6
BAX	F- GCATCCACCAAGAAGCTGAG<br>R- CCGCCACTCGGAAAAAGAC	130	XM_002701934.1
BCL2	F- ATGTGTGTGGAGAGCGTCA<br>R- AGAGACAGCCAGGAGAAATC	182	NM_001166486.1
SIRT1	F-AGCAGAAGAGGCGAGGGA<br>R-CCCTCTCAGCCGCCACTA	110	XM_015104377.4
PGC1α	F-GACTTGTGCAACCAGGACTC<br>R-GGCATGGAGGAAGGACTAGC	371	NM_001285631.1
ATP5F1B	F-ACTATGCCGCTCAAGCATCT<br>R-TACGGTGCTCTCACCCAAATG	191	NM_001686.4
ATP5FC1	F-CCAGCAAGAATGCTTCCGAAAT<br>R-AGACGAGAATGCTGAAGGCAA	148	XM_012188172.4
ATP6V1G	F-GGCGATTTGGCTGAGGTAGG<br>R-GCCTCCGGTTCTTTCGCTTG	167	XM_015092959.3
NDUFA1	F-CGCGCATCCACAGGTTCAGT<br>R-GTTTACTCCAGAGACGCGCCTA	104	NM_004541.4
NDUFA2	F-ACTTCATTGAGAAACGCTATGTGG<br>R-GCGAATCAGATCATCTTTAGACAGC	376	XM_012178619.5
NDUFA4	F-CAGGTGCGGTGAGTGCTTA<br>R-TGAAGAGGGGAATCAAGCTAGG	137	XM_004007753.4
NDUFV1	F-CTGGGACAACCTCCTTGCTG<br>R-TTTCACAATGTCTGTCGAGCG	166	XM_027959273.3
UQCRH	F-CTGAAGTGGAATAGGTGCCG<br>R-CAGCATCCTTTGCTCGTCCT	89	NM_001145187.2
CMC2	F-GTCCAGTCCGCGCGTTTG<br>R-GATGGAACACAGCAGAGCAAC	134	XM_042231484.1
COX15	F-TTGGGCACTGTTTATAGCATGCAG<br>R-CCAGACTCTGTCAACCTGTGT	123	XM_027960444.3
COX5A	F-TCCAGTCACTTCGCTGCTACTCC<br>R-AGTGTGTTCATCCCTTTACGCAAC	127	XM_027957175.2
NOX4	F-GGTTAAACACCTTTGCCTGCTTAT<br>R-AGCATCTGGTGGAGGTAGTGA	111	XM_012101598.4
NMNAT1	F-GGTGAAGCCGTGGGTTACAG<br>R-AAAGGACCCACAAGCAAGGAG	246	XM_004013736.5
NAMPT	F-CCCATTTTTCTCCTTCCTCGCA<br>R-TTTGCTTGTGTTGGGTGGGTA	166	XM_004007842.5
MFN1	F-GTTGTTGGAGGAGTGGTGTGGAAG<br>R-TCTGGTTCATGGCGGCGATTTC	301	XM_004013713.5
OPA1	F-GAACGCAGCATTGTTACAGACTTGG<br>R-AGCCTGTTGTTCAACTGACTCTCG	363	XM_027957232.2
EXTL3	F-CAGGCCATCCGAGATATGGT<br>R-TTAACCCATGCAAGACCCCC	969	XM_060409569.1
BMP15	F-AGCATGATGGGCCTGAAAGT	136	NM_001114767.2

	R-ACCCGAGGACATACTCCCTT		
GJA1	F-TGCAAAAGAGATCCCTGCCC	103	XM_004011159.5
	R-GAGACACCAACGACACGACA		
CYP11A1	F-CTGCGGAAGGAGGTTCTGAATGC	126	NM_001093789.1
	R-GGTCACGGAGATAGGGTGGAGTC		
STAR	F-GCGACCAAGAGCTTGCCTATATCC	94	NM_001009243.1
	R-CTCTCCTTCTTCCAGCCCTCCTG		
3βHSD	F-ATCCACACCAGCACCATAG	144	XM_012183658.2
	R-TTCCAGCACAGCCTTCTC		
NPPA	F-AAATCCCGTGTATGGCTCTG	92	NM_100294648
	R-CATCTTCTAAAGGCATCTTGTCC		
NPPB	F-ACGCTCCTGCTTCTCCTCTTCT	112	NM_100294642
	R-GGTCCAACAGCTCCTGTAACCC		
P450arom	F-TGGTGTCCGAAGTTGTGC	102	NM_101119602
	R-GACCTGGTATTGAGGATGTG		
P450scc	F-TGGCTCCAGAGGCAATAA	148	NM_100048994
	R-TCAAAGGCAAAGCGAAAC		
ULK1	F-GGACGAGATGTTCCACCGC	221	XM_027956386.2
	R- GAGAAGGTCCAGTCCTTTCGG		
LKB1	F-GTGAAGGAGGTGCTGGACTC	107	XM_004008774.3
	R-TCCTTCTTCACGTTGGCCTC		
AMPK	F-GTTCCTGGAGAAAGATGGCGA	122	XM_018065500.1
	R-TTTGCCAACCTTCACTTTGCC		
mTOR	F-TAAGAAAACGGGGACCACGG	282	NM_001285748.1
	R-CAAGAGAAGTGCTGTGGGCA		
RAPTOR	F-CACGATGGGTGTGGAGAACA	335	XM_004013082.4
	R-GGTGCTCAGCTGGCATGTA		
AMH	F-		
	R-		
AMHR	F-		
	R-		

Table S3 Details of specific antibody used for Western blot in the experiment

Antibodies	Cat NO.	Source	Dilution	Observed band (KDa)
PARP1	A0942	ABclonal (Wuhan, China)	1: 1000	89/113
PCNA	ab18197	Abcam (MA, USA)	1:500	29
BAX	50599-2-Ig	Protein Tech (IL, USA)	1:2000	24
BCL2	AF6139	Affinity (OH, USA)	1:1000	26
SIRT1	13161-AP	Protein Tech (IL, USA)	1:1000	110-130

CAT	21260-1-AP	Protein Tech (IL, USA)	1:2000	60
SOD2	24127-1-AP	Protein Tech (IL, USA)	1:2000	25
PGC1 α	ab106834	Abcam (MA, USA)	1:500	91
NAMPT	P43490	ZENBIO (Chengdu, China)	1:1000	56
CYC (cytochrome c)	10993-A-AP	Protein Tech (IL, USA)	1:2000	12
MFN1	DF7543	Affinity (OH, USA)	1:2000	84
OPA1	DF8587	Affinity (OH, USA)	1:2000	112
FIS1	DF12005	Affinity (OH, USA)	1:2000	17
STAR	bs3570R	Bioss (Beijing, China)	1:1000	32
CYP11A1	bs1099R	Bioss (Beijing, China)	1:1000	57
CYP19A1	DF6884	Affinity (OH, USA)	1:2000	58
AMPKα1/2	MCA2673GA	Bio-Rad (CA, USA)	1:1000	65
P-AMPKα (Thr172)	AF3423	Affinity (OH, USA)	1:2000	65
ULK1	DF7588	Affinity (OH, USA)	1:1000	115
P-ULK1 (ser566)	DF7587	Affinity (OH, USA)	1:1000	115
mTOR	2983	CST (MA, USA)	1:1000	245
P-mTOR (ser2448)	5536	CST (MA, USA)	1:1000	245
ACTB	bs-0061R	Bioss (Beijing, China)	1:5000	42
Pierce goat anti-rabbit Igg	31460	Pierce (OH, USA)	1:10000	-

(−): absent.

Table S4 Analysis of birth weight and body size index of sheep with high and low prolificacy

Items	High prolificacy group (HP)	Low prolificacy group (LP)	*P*-value
Body height/cm	71.67±1.881	71.75±1.978	>0.999

Body length/cm	63.93±5.817	68.35±4.293	0.202
Chest circumference/cm	88.67±4.130	95.02±2.378	0.014
Pipe circumference/cm	8.567±0.741	8.267±0.629	0.506
Shiri wide/cm	16.95±2.173	17.40±1.626	0.716

Results were analyzed using one-way ANOVA. Data were presented as mean ± SEM (n = 3). Means in the same row with different superscript letters are significantly different ($P < 0.05$). Means in the same row with no letters are not significantly different ($P > 0.05$). The same as flowing.

Table S5 Blood hormone concentration during estrus cycle of sheep with high and low prolificacy

Items	Groups	Day 1 of the estrous cycle	Day 2 of the estrous cycle	Day 3 of the estrous cycle	Day 4 of the estrous cycle	Day 7 of the estrous cycle	Day 11 of the estrous cycle	Day 15 of the estrous cycle
E2(pg/mL)	HP	24.69±3.88	23.19±4.26	24.13±3.92	23.74±3.60	24.60±3.74	26.61±4.20	24.28±3.51
	LP	21.51±1.74	22.74±2.58	22.48±0.91	21.87±2.38	21.09±1.70	23.09±2.11	22.6±3.71
P4(ng/mL)	HP	6.06±0.50	6.20±0.53	6.55±0.54	5.99±0.69	6.18±0.54	6.15±0.40	5.98±0.68
	LP	5.37±0.71	5.36±0.94	5.42±0.72	5.30±0.62	5.65±0.71	5.31±0.85	5.57±0.43
LH(mIU/mL)	HP	7.12±0.24	7.48±0.27	6.95±0.51	7.45±0.37	6.45±0.47	7.89±0.43	9.95±0.89
	LP	7.78±1.06	8.12±1.26	7.90±1.02	7.99±1.23	7.91±0.86	8.01±1.09	8.98±0.46
FSH(mIU/mL)	HP	62.69±10.93	59.20±13.68	65.55±10.44	61.41±11.28	63.28±10.06	65.74±10.09	51.59±5.89
	LP	64.91±7.36	66.49±8.97	71.95±6.13	64.62±8.84	62.45±8.79	67.77±8.35	77.67±2.82
GnRH(mIU/mL)	HP	54.92±5.74	52.91±5.74	53.73±5.85	56.96±5.40	55.63±7.78	60.28±5.89	57.18±4.46
	LP	56.70±5.17	55.53±6.67	59.15±3.88	54.79±5.35	57.44±4.67	57.39±6.40	62.57±6.14
FS(ng/mL)	HP	8.09±1.04	7.80±1.04	8.97±1.00	8.26±1.20	7.96±1.37	8.03±0.81	8.38±0.98
	LP	7.89±0.90	8.11±1.20	8.05±0.99	7.28±1.27	8.77±1.00	7.52±1.08	7.89±0.59

Table S6 Blood LH concentration within 3 h after estrus of sheep with high and low prolificacy

Times \ Groups	HP (mIU/mL)	LP (mIU/mL)
0~ 30 min	10.131±0.179	9.186±0.202

30~90 min	10.41±0.208[a]	9.027±0.255[b]
90-150 min	10.187±0.157[a]	8.782±0.441[b]
Mean value	10.254±0.252[a]	8.929±0.38[b]

Table S7 Blood FSH concentration within 3 h after estrus of sheep with high and low prolificacy

Groups / Times	HP (mIU/mL)	LP (mIU/mL)
0~ 30 min	52.055±0.465[b]	78.455± 0.785[a]
30~90 min	53.79±1.86[b]	79.063±0.992[a]
90-150 min	53.77±2.626[b]	78.75±2.194[a]
Mean value	53.64±2.378[b]	78.488±1.653[a]

Table S8 Blood GnRH concentration within 3 h after estrus of sheep with high and low prolificacy

Groups / Times	HP (mIU/mL)	LP (mIU/mL)
0~ 30 min	57.305±0.125[b]	66.33±1.35[a]
30~90 min	58.33±1.707[b]	67.58±0.247[a]
90-150 min	58.893±2.626[b]	68.253±1.927[a]
Mean value	58.668±2.191[b]	67.443±1.792[a]

Table S9 Blood FS concentration within 3 h after estrus of sheep with high and low prolificacy

Groups / Times	HP (mIU/mL)	LP (mIU/mL)
0~ 30 min	8.201±0.616	8.677±0.0145
30~90 min	7.924±0.777	9.264±0.501
90-150 min	7.619±0.523[b]	9.002±0.227[a]
Mean value	7.882±0.579[b]	9.042±0.437[a]

Table S10 Blood E2 concentration within 3 h after estrus of sheep with high and low prolificacy

Groups / Times	HP (mIU/mL)	LP (mIU/mL)
0~ 30 min	24.91±0.63	23.175±0.575
30~90 min	25.613±0.164[a]	22.827±0.799[b]
90-150 min	24.603±1.017	22.817±1.056
Mean value	24.848±0.862[a]	22.767±0.938[b]

Table S11 Blood P4 concentration within 3 h after estrus of sheep with high and low prolificacy

Times \ Groups	HP (mIU/mL)	LP (mIU/mL)
0~ 30 min	5.996±0.012	5.431±0.14
30~90 min	5.935±0.092[a]	5.490±0.141[b]
90-150 min	5.927±0.061[a]	5.572±0.157[b]
Mean value	5.93±0.077[a]	5.526±0.153[b]

Table S12 Slaughter traits of sheep with high and low prolificacy

Items	High prolificacy group (HP)	Low prolificacy group (LP)	*P*-value
Initial body weight /kg	54.60±6.904	56.70±6.699	0.634
Carcass weight/kg	22.30±3.567	25.58±4.266	0.216
Dressing percentage/%	41.347±2.067	44.326±2.359	0.094

Table S13 Organ weight and its index of sheep with high and low prolificacy

Items	High prolificacy group (HP)	Low prolificacy group (LP)	*P*-value
Pituitary weight/g	1.189±0.165	0. 928±0.073	0.111
Ovary weight/g	1.554±0.243	1.110±0.243	0.206
Uterus weight/g	127.2±34.026	115.4±19.265	0.514
Coefficients of pituitary/%	0.057±0.008	0.044±0.003	0.111
Coefficients of ovary/%	0.074±0.012	0.053±0.034	0.206
Coefficients of uterus/%	6.088±1.628	5.522±0.922	0.514

Table S14 Alignment of sequenced reads processing and statistics of raw data in the sheep ovarian cells with high and low prolificacy

Sample	Raw Reads	Clean Reads	Q20 (%)	Q30 (%)
LP_B_GCs1	49943702	43027600	94.50	89.75
LP_B_GCs2	54143454	50402144	96.44	91.95
LP_B_GCs3	41571578	38808298	95.62	88.87
HP_B_GCs1	50664470	48138166	97.23	93.06
HP_B_GCs2	48260804	45495728	97.12	92.68
HP_B_GCs3	61929460	56313088	95.93	91.00
LP_B_TCs1	43032220	42820692	98.00	94.21
LP_B_TCs2	50218810	49989756	98.05	94.33
LP_B_TCs3	46291278	46056470	98.17	94.61
HP_B_TCs1	52654686	52376346	97.48	93.12
HP_B_TCs2	53055374	52788328	97.86	93.99
HP_B_TCs3	57880022	57588094	97.61	93.43

Table S15 Alignment of the clean reads mapping to the reference genome in sheep ovarian cells with high and low prolificacy

Sample	Total mapped	Uniquely mapped	Multi Mapped reads
LP_B_GCs1	38654832 (88.23%)	35285236 (80.54%)	3369596 (7.69%)

LP_B_GCs2	42345706 (84.02%)	35781414 (70.99%)	6564292 (13.02%)
LP_B_GCs3	34488072 (88.87%)	31640580 (81.53%)	2847492 (7.34%)
HP_B_GCs1	42399504 (88.08%)	39124026 (81.27%)	3275478 (6.8%)
HP_B_GCs2	40212612 (88.39%)	35660320 (78.38%)	4552292 (10.01%)
HP_B_GCs3	47071908 (83.59%)	41761760 (74.16%)	5310148 (9.43%)
LP_B_TCs1	39388336 (91.98%)	36696578 (85.7%)	2691758 (6.29%)
LP_B_TCs2	46152864 (92.32%)	42588338 (85.19%)	3564526 (7.13%)
LP_B_TCs3	42864520 (93.07%)	38963292 (84.6%)	3901228 (8.47%)
HP_B_TCs1	41853518 (92.48%)	38058016 (84.1%)	3795502 (8.39%)
HP_B_TCs2	47870712 (91.4%)	43945280 (83.9%)	3925432 (7.49%)
HP_B_TCs3	48393986 (91.68%)	44383688 (84.08%)	4010298 (7.6%)

Table S16 Significantly enriched KEGG pathway by differential metabolites

Map ID	Map Title	*P*-value
oas05140	Leishmaniasis	0.0002605
oas04921	Oxytocin signaling pathway	0.001129
oas05146	Amoebiasis	0.001331
oas04923	Regulation of lipolysis in adipocytes	0.001549
oas04931	Insulin resistance	0.002876
oas04750	Inflammatory mediator regulation of TRP channels	0.009621
oas00500	Starch and sucrose metabolism	0.01072
oas05230	Central carbon metabolism in cancer	0.01072
oas05415	DIABETIC CARDIOMYPATHY	0.01186
oas05165	Human papillomavirus infection	0.01302
oas05323	Rheumatoid arthritis	0.01302
oas04726	Serotonergic synapse	0.01368
oas00563	Glycosylphosphatidylinositol (GPI)-anchor biosynthesis	0.01733
oas04912	GnRH signaling pathway	0.02589
oas05163	Human cytomegalovirus infection	0.02589
oas04666	Fc gamma R-mediated phagocytosis	0.03438
oas05143	African trypanosomiasis	0.03438
oas04730	Long-term depression	0.0386
oas04217	Necroptosis	0.0428
oas00590	Arachidonic acid metabolism	0.04472
oas04625	C-type lectin receptor signaling pathway	0.04698
oas04664	Fc epsilon RI signaling pathway	0.04698
oas04917	Prolactin signaling pathway	0.04698
